# Policy aware fusion of educational and economic indicators for public health curriculum optimization

**DOI:** 10.3389/fpubh.2026.1771114

**Published:** 2026-08-06

**Authors:** Ye Wang

**Affiliations:** School of Foreign Languages, Zhejiang University of Finance & Economics Dongfang College, Haining, Zhejiang, China

**Keywords:** course passing prediction, educational data mining, learning effectiveness prediction, policy aware indicator fusion, public health curriculum optimization, uncertainty aware modeling

## Abstract

**Introduction:**

The optimization of public health curricula requires the effective integration of heterogeneous educational, socio economic, behavioral, and course context indicators under policy related constraints. Traditional curriculum design approaches often rely on static rules or isolated educational measurements, making it difficult to capture dynamic learner course interactions, policy consistency, and uncertainty in real world online learning environments. To address these challenges, this study proposes a policy aware framework, termed the Policy Driven Indicator Integrator, for supporting public health curriculum optimization through learning effectiveness prediction.

**Methods:**

The proposed framework integrates multiple sources of learner course information into a shared latent representation while incorporating policy constrained regularization, event driven temporal modeling, and uncertainty aware refinement. The Manifold Constrained Optimizer learns policy consistent latent representations from educational, socio economic, behavioral, quiz, and assignment indicators; the Event Driven Curriculum Planner captures temporal learning dynamics across course weeks; and the Uncertainty Propagation Forecaster improves robustness under incomplete or noisy learning records. The Event Segmentation Alignment strategy refines curriculum related representations by aligning temporal, participation, and policy dimensions within a feasibility constrained optimization process. To quantitatively evaluate the framework, curriculum optimization is formulated as a supervised course learning effectiveness prediction task, where course passing status serves as an operational proxy for successful learning outcomes. Experiments on OULAD and the HarvardX MITx Person Course Academic Year 2013 De Identified Dataset demonstrate that the proposed method consistently outperforms representative statistical learning, machine learning, gradient boosted tree, sequence learning, deep tabular learning, and Transformer based tabular baselines in terms of Accuracy, Macro F1, AUROC, and AUPRC.

**Results and Discussion:**

The results show that policy aware indicator fusion, event driven modeling, and uncertainty propagation provide complementary benefits for robust public health curriculum optimization. This work offers a systematic and data driven approach for improving curriculum decision support in public health education while maintaining consistency with policy aware educational objectives.

## 1 Introduction

Public health curriculum optimization is an important task for preparing learners to understand health systems, respond to population level challenges, and apply evidence informed knowledge in practical contexts. In online and data supported public health education, curriculum optimization requires more than the selection of course topics. It also requires the systematic analysis of learner characteristics, course context information, behavioral engagement, assessment performance, and policy related constraints (1). These heterogeneous indicators jointly influence learning effectiveness and provide useful evidence for improving curriculum organization, resource allocation, and instructional support.

Traditional curriculum design approaches often rely on predefined rules, expert experience, or static instructional frameworks. Although these approaches provide useful domain guidance, they are usually limited in their ability to capture dynamic learner course interactions and evolving educational demands. In particular, rule based curriculum planning may overlook temporal learning behaviors, differences in learner engagement, and uncertainty caused by incomplete or noisy platform records (2). As a result, such approaches may produce curriculum decisions that are interpretable but insufficiently adaptive to real learning environments.

To improve adaptability, data driven methods have been increasingly introduced into educational modeling and curriculum analysis. Statistical learning and traditional machine learning methods can identify relationships between learner course features and learning outcomes, thereby supporting more evidence based curriculum decisions. Models such as logistic regression, random forest, and gradient boosted decision trees are effective for structured educational data and offer useful baselines for learning effectiveness prediction. However, these methods often depend heavily on manually designed features and may have limited ability to model temporal learning dynamics or higher order interactions among heterogeneous indicators (3).

Recent deep learning and Transformer based tabular learning methods further improve the modeling of complex educational data. Sequence models can capture weekly learning behaviors, while deep tabular models can learn nonlinear relationships among demographic, registration, activity, quiz, and assignment features. Despite these advantages, existing methods still face several limitations when applied to public health curriculum optimization. Many models focus mainly on predictive accuracy and do not explicitly incorporate policy aware constraints or curriculum related domain knowledge. Temporal learning dynamics are often modeled independently from policy and participation dimensions, which weakens the connection between prediction and curriculum decision support. Uncertainty in learner course records, such as missing activity logs or noisy assessment information, is not always systematically propagated into the modeling process (4).

To address these limitations, this study proposes a policy aware indicator fusion framework for public health curriculum optimization. The proposed framework, termed the Policy Driven Indicator Integrator, integrates educational, socio demographic, behavioral, assessment, and course context indicators into a shared latent modeling space. It incorporates policy constrained representation learning, event driven temporal curriculum modeling, and uncertainty aware refinement to improve both predictive performance and curriculum interpretability. Instead of treating curriculum optimization as a purely conceptual design problem, this study formulates it as a supervised learning effectiveness prediction task, where course passing status serves as an operational proxy for successful learning outcomes. This formulation enables quantitative evaluation while remaining consistent with the goal of improving public health curriculum decision support.

The proposed framework contains three main components. The Manifold Constrained Optimizer learns policy consistent latent representations from heterogeneous learner course indicators. The Event Driven Curriculum Planner captures temporal learning dynamics by modeling weekly learner behaviors and event related curriculum patterns. The Uncertainty Propagation Forecaster estimates and propagates uncertainty from input indicators to enhance robustness under incomplete or noisy data. The Event Segmentation Alignment strategy refines curriculum related representations by aligning temporal, participation, and policy dimensions within a feasibility constrained optimization process.

We summarize our contributions as follows:

- We propose a policy aware indicator fusion framework for public health curriculum optimization, integrating heterogeneous learner course indicators into a shared policy constrained latent representation.
- We formulate curriculum optimization as a supervised course learning effectiveness prediction task, using course passing status as an operational proxy for evaluating curriculum related learning outcomes.
- We design event driven temporal modeling and uncertainty aware refinement modules to capture weekly learning dynamics and improve robustness under incomplete or noisy learning records.
- We conduct experiments on OULAD and the HarvardX MITx Person Course Academic Year 2013 De Identified Dataset, demonstrating consistent improvements over statistical learning, traditional machine learning, gradient boosted tree, sequence learning, deep tabular learning, and Transformer based tabular baselines.

To ensure coherence between the declared educational problem, the proposed method, and the experimental strategy, this study does not treat curriculum optimization as a multimodal perception problem or as an image based classification task. Instead, the research problem is defined within data supported public health education: heterogeneous learner course records are used to estimate learning effectiveness, policy aware constraints are used to preserve curriculum related objectives, temporal learning events are used to capture pedagogical progression, and uncertainty modeling is used to improve the reliability of curriculum decision support. The scientific contribution of this work lies not in applying multimodal vision language learning to education, but in developing an operational framework that connects educational indicators, policy aware modeling, supervised learning effectiveness prediction, and curriculum interpretation within a public health education context.

## 2 Related work

### 2.1 Curriculum theory and public health education

Curriculum is not merely a technical arrangement of course contents or a collection of measurable learning indicators. From an educational perspective, curriculum also reflects assumptions about what knowledge is considered valuable, whose experiences are represented, how learning opportunities are distributed, and how institutional goals are translated into pedagogical practice (5). In public health education, this issue is especially important because curriculum knowledge is closely related to population health, social inequality, institutional preparedness, health literacy, and collective responsibility (6). Curriculum optimization should not be understood as a purely computational search for higher predictive performance, but as a decision support process situated within educational, social, and policy contexts. From the perspective of curriculum epistemology, the indicators used in this study provide only a partial and operational representation of curriculum implementation. Educational background, socio demographic context, online engagement, quiz performance, assignment completion, and course outcomes do not exhaust the meaning of curriculum. Instead, they describe observable traces of how learners encounter and respond to curriculum structures in online learning environments. This view is consistent with curriculum theory that regards curriculum as a complex educational conversation rather than a fixed technical object. The proposed framework therefore treats data driven modeling as a way to support curriculum reflection rather than as a replacement for curriculum interpretation. Public health curricula also involve political and ideological considerations. Decisions about curriculum priorities may reflect different views on equity, accessibility, resource allocation, community preparedness, professional competence, and the balance between individual responsibility and collective health. Critical curriculum scholarship has emphasized that curriculum knowledge is never fully neutral, because it is shaped by social power, institutional interests, and cultural selection (7). These issues cannot be resolved by prediction models alone. In this study, policy aware constraints are introduced to prevent curriculum related modeling from being guided only by unconstrained predictive accuracy. The purpose of these constraints is to make the optimization process more consistent with public health education objectives and to provide a formal mechanism for incorporating policy relevant concerns into learning effectiveness prediction. Pedagogical mediation is another necessary dimension of curriculum optimization. Model outputs should not be interpreted as automatic curriculum prescriptions. Rather, predicted learning effectiveness, uncertainty estimates, and event aligned representations should be used as evidence for teachers, curriculum designers, and educational administrators to examine learner engagement, identify possible barriers, and adjust instructional support. This position is consistent with critical pedagogical perspectives that emphasize teacher judgment, learner agency, and reflective educational practice (8). In this sense, the proposed framework functions as a pedagogical decision support tool. It provides quantitative evidence for curriculum reflection while leaving final curriculum judgment to educators and policy stakeholders who understand the local teaching context.

### 2.2 Heterogeneous indicator modeling in public health education

Public health education is influenced by multiple types of indicators, including learner background, course context, learning behavior, assessment performance, and policy related requirements. These indicators provide complementary information for understanding learning effectiveness and improving curriculum organization. In online and data supported learning environments, demographic attributes, registration records, platform interactions, quiz performance, assignment completion, and course outcomes can be jointly analyzed to reveal how learners engage with course content and how curriculum structures affect learning results (9). The integration of heterogeneous indicators is particularly important for public health curriculum optimization because public health education involves both knowledge acquisition and the ability to respond to complex social and institutional challenges. Educational indicators describe learners' academic progress and engagement patterns, while socio demographic and course context indicators provide additional information about learner diversity, participation conditions, and resource related differences. When these indicators are modeled together, they can support more reliable learning effectiveness prediction and provide evidence for curriculum refinement (10). However, heterogeneous indicator modeling also introduces several challenges. Different indicators may have different scales, formats, temporal structures, and missing value patterns. Static feature aggregation may ignore weekly learning dynamics, while purely predictive models may fail to preserve curriculum related interpretability. Learner course records collected from online platforms are often incomplete or noisy, which can reduce the reliability of curriculum decision support. These challenges motivate the development of a unified framework that can fuse heterogeneous indicators, preserve policy related constraints, and remain robust under data uncertainty (11).

### 2.3 Policy aware curriculum optimization

Policy aware curriculum optimization aims to align curriculum decisions with educational objectives, institutional requirements, and broader public health priorities. In public health education, curriculum design should not only improve learning outcomes but also reflect policy relevant concerns such as equity, accessibility, resource allocation, health literacy, and preparedness for population level health challenges. Curriculum optimization requires a modeling process that can incorporate domain knowledge and policy related constraints rather than relying only on unconstrained predictive performance (12). Traditional policy driven curriculum development often depends on expert knowledge, predefined competency frameworks, and manual curriculum review. These approaches are useful for maintaining interpretability and ensuring consistency with educational goals. Nevertheless, they may be limited in scalability and responsiveness when learner behavior and course participation patterns change rapidly. As online learning platforms generate increasingly detailed learner course records, policy aware curriculum optimization can benefit from data driven modeling while still preserving domain constraints (13). A key issue is how to connect policy awareness with quantitative learning analytics. If policy requirements are treated only as external descriptions, they may not directly influence model training or prediction. Conversely, if models are optimized only for predictive accuracy, they may produce results that are difficult to interpret or misaligned with curriculum goals. A policy constrained representation learning strategy provides a possible solution by embedding heterogeneous learner course indicators into a shared latent space while regularizing the representation according to policy related constraints. This allows curriculum optimization to remain both data driven and policy consistent (14).

### 2.4 Learning effectiveness prediction and educational data mining

Learning effectiveness prediction is a central task in educational data mining and learning analytics. It aims to predict learners' academic outcomes from observed learner course features, such as demographic information, registration status, learning activities, assessment scores, and temporal engagement patterns. Course passing status prediction is especially useful for curriculum optimization because it provides an operational measure of whether learners successfully complete a course under a given curriculum and instructional setting (15). Traditional statistical and machine learning methods, such as logistic regression, random forest, and gradient boosted decision trees, have been widely used for structured educational data. These methods can capture important relationships between learner course features and course outcomes, and they often provide strong baselines for offline evaluation. However, they may depend on manually constructed features and may not fully model temporal learning behavior, especially when weekly engagement patterns and assessment trajectories are important for predicting learning outcomes (16). Deep learning methods have extended learning effectiveness prediction by modeling nonlinear feature interactions and temporal dependencies. Sequence models can process ordered weekly behavior records, while deep tabular models and Transformer based architectures can learn complex relationships among structured learner course indicators. Despite their effectiveness, these methods often lack explicit policy aware constraints and may be sensitive to missing or noisy learning records. For public health curriculum optimization, it is therefore necessary to combine predictive modeling with policy constrained representation learning, event driven temporal modeling, and uncertainty aware refinement (17).

### 2.5 Uncertainty aware and event driven curriculum modeling

Online learning records are rarely complete or perfectly reliable. Learners may have irregular platform interactions, missing activity logs, incomplete assessment records, or inconsistent participation patterns. If these uncertainties are ignored, curriculum related predictions may become unstable and less useful for decision support. Uncertainty aware modeling addresses this issue by estimating the variability of input indicators and propagating it into the prediction or optimization process. This is particularly important for public health education, where curriculum decisions may affect resource planning, learner support, and instructional adjustment (18). Event driven modeling provides another important perspective for curriculum optimization. Learning behavior changes over time, and weekly activity patterns can reflect how learners respond to course content, assessments, deadlines, and instructional interventions. By modeling temporal events and aligning them with curriculum representations, an event driven approach can capture dynamic learning processes that static feature models may overlook. This is especially relevant when curriculum optimization is evaluated through course passing status prediction, because successful completion often depends on sustained engagement across the course timeline (19). Existing approaches often consider uncertainty modeling, temporal modeling, and curriculum alignment as separate components. In contrast, a unified framework can combine these aspects to support robust public health curriculum optimization. By integrating policy constrained latent representation learning, event driven curriculum planning, and uncertainty propagation, the proposed approach is designed to improve learning effectiveness prediction while maintaining consistency with curriculum and policy objectives (20).

## 3 Method

### 3.1 Overview

This section introduces the proposed *Policy Driven Indicator Integrator* for policy aware public health curriculum optimization. Consistent with the experimental setting, curriculum optimization is evaluated through a supervised course learning effectiveness prediction task, where course passing status is used as an operational proxy for successful learning outcomes. The framework integrates heterogeneous learner course indicators, including educational and learner background features, socio demographic and course context information, online learning behavior, quiz records, and assignment records. These indicators jointly describe learner profiles, enrollment context, behavioral engagement, formative assessment performance, and coursework completion patterns. The proposed methodology is organized into three principal components. Section 3.2 formulates public health curriculum optimization as a policy aware learning effectiveness prediction problem. It defines the learner course input space, binary passing status label, policy constrained latent manifold, temporal behavior sequence, and uncertainty propagation mechanism. This formulation establishes the mathematical foundation for connecting heterogeneous learner course modeling with curriculum optimization.

Section 3.4 details the *Policy Driven Indicator Integrator*, which consists of three core modules: the *Manifold Constrained Optimizer*, the *Event Driven Curriculum Planner*, and the *Uncertainty Propagation Forecaster*. The *Manifold Constrained Optimizer* learns policy consistent latent representations from heterogeneous learner course indicators. The *Event Driven Curriculum Planner* captures weekly learning dynamics by modeling temporally ordered learning behaviors and event aware curriculum representations. The *Uncertainty Propagation Forecaster* estimates the influence of missing, noisy, or unstable learner course records and introduces uncertainty aware regularization to improve robustness. Section 3.5 introduces the *Event Segmentation Alignment* strategy, which further refines the representations produced by the *Policy Driven Indicator Integrator*. This strategy aligns segmented weekly learning events with curriculum related latent representations by integrating temporal, participation, and policy dimensions. Feasibility manifold refinement is used to preserve curriculum and policy consistency, while uncertainty guided alignment reduces the influence of unreliable event records. Together, these components provide a unified framework for course passing status prediction and policy aware public health curriculum optimization.

### 3.2 Preliminaries

This subsection establishes the mathematical formulation for policy aware public health curriculum optimization. Consistent with the experimental setting, curriculum optimization is evaluated through a supervised learning effectiveness prediction task, where the course passing status is used as an operational proxy for successful learning outcomes. The objective is to model heterogeneous learner course indicators under policy related constraints and to provide a foundation for the subsequent Policy Driven Indicator Integrator.

Let X denote the learner course input space. For each learner course instance, the input vector is represented as


$$\begin{array}{l} {\text{x}}_{s}=\left[{\text{x}}_{s}^{edu},{\text{x}}_{s}^{ctx},{\text{x}}_{s}^{beh},{\text{x}}_{s}^{quiz},{\text{x}}_{s}^{assign}\right], \end{array}$$ (1)

where *s* indexes a learner course sample. Here, ${\text{x}}_{s}^{edu}$ denotes educational and learner background indicators, ${\text{x}}_{s}^{ctx}$ denotes socio demographic and course context indicators, ${\text{x}}_{s}^{beh}$ denotes online learning behavior indicators, ${\text{x}}_{s}^{quiz}$ denotes quiz related indicators, and ${\text{x}}_{s}^{assign}$ denotes assignment related indicators. These indicators correspond to the heterogeneous feature groups used for course passing status prediction.

Let *y*_*s*_ ∈ {0, 1} denote the binary learning outcome of sample *s*, where *y*_*s*_ = 1 indicates that the learner passes the course and *y*_*s*_ = 0 indicates that the learner does not pass the course. The dataset is defined as


$$\begin{array}{l} S={\left\{\left({\text{x}}_{s},y_{s}\right)\right\}}_{s=1}^{N}, \end{array}$$ (2)

where *N* is the number of learner course samples. The goal is to learn a prediction function


$$\begin{array}{l} f_{\theta }:X\to \left[0,1\right], \end{array}$$ (3)

where *f*_θ_(**x**_*s*_) estimates the probability that learner course sample *s* leads to a passing outcome.

To support policy aware curriculum optimization, heterogeneous indicators are first projected into a shared latent representation. Let


$$\begin{array}{l} {\text{z}}_{s}=g_{\theta }\left({\text{x}}_{s}\right),\;{\text{z}}_{s}\in {\mathbb{R}}^{d_{z}}, \end{array}$$ (4)

where *g*_θ_(·) is the representation learning function and *d*_*z*_ is the latent embedding dimension. The latent representation **z**_*s*_ is expected to preserve predictive information from heterogeneous learner course indicators while satisfying curriculum and policy related constraints.

Policy related constraints are denoted by P. They encode requirements associated with curriculum consistency, resource awareness, participation balance, learning progression, and other policy relevant educational objectives. In the latent space, these constraints are represented as inequality functions:


$$\begin{array}{l} P=\left\{p_{m}\left({\text{z}}_{s}\right)\leq \sigma_{m}|m=1,2,\ldots ,M\right\}, \end{array}$$ (5)

where *p*_*m*_(·) is the *m*-th policy related constraint function, σ_*m*_ is the corresponding threshold, and *M* is the number of constraints. A policy regularized feasible region is then defined as


$$\begin{array}{l} M=\left\{{\text{z}}_{s}\in {\mathbb{R}}^{d_{z}}|p_{m}\left({\text{z}}_{s}\right)\leq \sigma_{m},\;\forall m=1,2,\ldots ,M\right\}. \end{array}$$ (6)

Here, M denotes the feasible latent manifold that contains curriculum consistent learner course representations.

The supervised learning objective is formulated by combining prediction loss and policy aware regularization. For binary course passing status prediction, the empirical risk can be written as


$$\begin{array}{l} L_{pred}=-\frac{1}{N}\overset{N}{\underset{s=1}{\sum }}\left[y_{s}\log f_{\theta }\left({\text{x}}_{s}\right)+\left(1-y_{s}\right)\log\left(1-f_{\theta }\left({\text{x}}_{s}\right)\right)\right]. \end{array}$$ (7)

To penalize violations of policy related constraints, the policy regularization term is defined as


$$\begin{array}{l} L_{pol}=\frac{1}{N}\overset{N}{\underset{s=1}{\sum }}\overset{M}{\underset{m=1}{\sum }}\max\left(0,p_{m}\left({\text{z}}_{s}\right)-\sigma_{m}\right). \end{array}$$ (8)

The basic policy aware optimization objective is therefore


$$\begin{array}{l} \underset{\theta }{\min}L_{pred}+\lambda L_{pol}, \end{array}$$ (9)

where λ controls the contribution of policy aware regularization.

To model temporal learning dynamics, each learner course instance may also contain a weekly behavior sequence:


$$\begin{array}{l} {\text{B}}_{s}=\left[{\text{b}}_{s,1},{\text{b}}_{s,2},\ldots ,{\text{b}}_{s,T}\right], \end{array}$$ (10)

where **b**_*s,t*_ denotes the learning behavior representation of learner *s* at relative course week *t*, and *T* is the maximum sequence length. These temporal records provide the basis for event driven curriculum planning by capturing changes in learner engagement, participation, and assessment behavior across the course timeline.

Uncertainty is introduced because learner course indicators may contain missing values, noisy activity records, or incomplete assessment information. Let Σ_**x**_ denote the covariance or uncertainty estimate of the input indicators. The corresponding latent uncertainty is represented as


$$\begin{array}{l} \Sigma_{\text{z}}=J_{g}\Sigma_{\text{x}}J_{g}^{⊤}, \end{array}$$ (11)

where *J*_*g*_ is the Jacobian matrix of the representation function *g*_θ_(·) with respect to the input indicators. In practice, this uncertainty term is incorporated into the training objective to improve robustness:


$$\begin{array}{l} \underset{\theta }{\min}L_{pred}+\lambda L_{pol}+\gamma \text{ Tr}\left(\Sigma_{\text{z}}\right), \end{array}$$ (12)

where Tr(·) denotes the trace operator and γ controls the influence of uncertainty aware regularization.

This formulation defines public health curriculum optimization as a policy aware learning effectiveness prediction problem. It establishes the latent feasible manifold, policy constrained optimization objective, temporal learner course representation, and uncertainty propagation mechanism used by the Policy Driven Indicator Integrator in the following subsections.

### 3.3 Policy constraints used in experiments

To make the policy aware optimization process more concrete and reproducible, this subsection specifies the policy constraints used in the experiments. In the proposed framework, policy constraints are not manually assigned labels or external administrative decisions. Instead, they are operationalized from observed learner course records, including weekly activity, assessment performance, course progression, learner participation, and missingness patterns. These constraints are used to regularize the latent representation so that the learned learner course embedding remains consistent with curriculum related objectives rather than being optimized only for prediction accuracy. Let **z**_*s*_ denote the latent representation of learner course sample *s*, and let ${\hat{q}}_{m}\left({\text{x}}_{s}\right)$ denote an observed policy related indicator computed from the original learner course features **x**_*s*_. Each policy constraint is implemented through a differentiable projection head *p*_*m*_(**z**_*s*_) that estimates the corresponding policy related indicator from the latent representation. The constraint violation is then measured by a hinge penalty:


$$\begin{array}{l} L_{pol}=\frac{1}{N}\overset{N}{\underset{s=1}{\sum }}\overset{M}{\underset{m=1}{\sum }}\max\left(0,p_{m}\left({\text{z}}_{s}\right)-\sigma_{m}\right), \end{array}$$ (13)

where σ_*m*_ is the threshold of the *m* th policy constraint. In the experiments, four policy constraints are considered: learning progression consistency, participation balance, assessment completion consistency, and data reliability. These constraints are computed from observed features in OULAD, HarvardX MITx, and the public health focused HarvardX MITx subset using the same normalization strategy. All continuous indicators are normalized to [0, 1] using statistics from the training split, and threshold values are selected from the training distribution to avoid information leakage.

In Table 1, ${\hat{r}}_{s,t}$ denotes the normalized activity intensity of learner course sample *s* at week *t*, *T* denotes the maximum number of course weeks, $n_{s}^{assess}$ denotes the number of valid assessment related records for sample *s*, $n_{max}^{assess}$ denotes the maximum possible number of assessment records in the corresponding course, and *D* denotes the number of observed input fields. The policy heads *p*_*m*_(**z**_*s*_) are trained jointly with the main prediction objective, while the observed indicators ${\hat{q}}_{m}\left({\text{x}}_{s}\right)$ provide the empirical basis for computing policy related violations. In this way, policy constraints are grounded in measurable course records and can be applied consistently across the datasets. These constraints are not intended to encode rigid institutional rules. Rather, they provide operational proxies for curriculum relevant principles that are commonly important in online public health education, including stable learning progression, sustained participation, assessment completion, and reliability of learner course evidence. The thresholds are determined only from the training split or set according to transparent curriculum motivated criteria. The experimental implementation avoids using test information while making the policy aware component explicit and reproducible.

**Table 1:** Policy constraints used in the experiments.

Policy constraint	Curriculum motivation	Observed features	Mathematical definition	Threshold setting
Learning progression consistency	Learners are expected to maintain a reasonable learning trajectory across course weeks rather than showing abrupt disengagement or highly unstable activity patterns.	Weekly activity counts, active week indicators, video or chapter access records, virtual learning environment interactions	$p_{1}\left({\text{z}}_{s}\right)=|{\hat{r}}_{s,T}-{\hat{r}}_{s,1}|+\frac{1}{T-1}\overset{T}{\underset{t=2}{\sum }}|{\hat{r}}_{s,t}-{\hat{r}}_{s,t-1}|$	σ_1_ is set to the 75th percentile of this indicator in the training split.
Participation balance	Curriculum decision support should not rely only on a single burst of participation. Learner engagement should be distributed across the course timeline.	Number of active days, active weeks, weekly interaction counts, forum participation, chapter access frequency	$p_{2}\left({\text{z}}_{s}\right)=1-\frac{\overset{T}{\underset{t=1}{\sum }}𝕀\left({\hat{r}}_{s,t}>0\right)}{T}$	σ_2_ = 0.50, requiring at least half of the observed course weeks to show valid participation when records are available.
Assessment completion consistency	Public health curriculum evaluation should consider whether learners complete formative or summative assessments consistently rather than only interacting with content passively.	Quiz attempts, assignment submissions, assessment scores, grade related indicators, certification related records	$p_{3}\left({\text{z}}_{s}\right)=1-\frac{n_{s}^{assess}}{n_{max}^{assess}}$	σ_3_ is set to the 75th percentile of the assessment incompletion rate in the training split.
Data reliability	Predictions should be cautious when learner course records contain excessive missingness or unstable observations, because unreliable records may weaken curriculum decision support.	Missing demographic values, missing activity records, missing assessment fields, abnormal activity counts, invalid grade values	$p_{4}(z_{s})=\frac{1}{D}\sum_{j=1}^{D}𝕀(x_{s,j}$ is missing or invalid)	σ_4_ = 0.20, allowing at most 20 percent unreliable observed fields after data curation.

Each constraint is computed from observed learner course features and incorporated into the policy regularization term.

### 3.4 Policy driven indicator integrator

**Figure 1:**
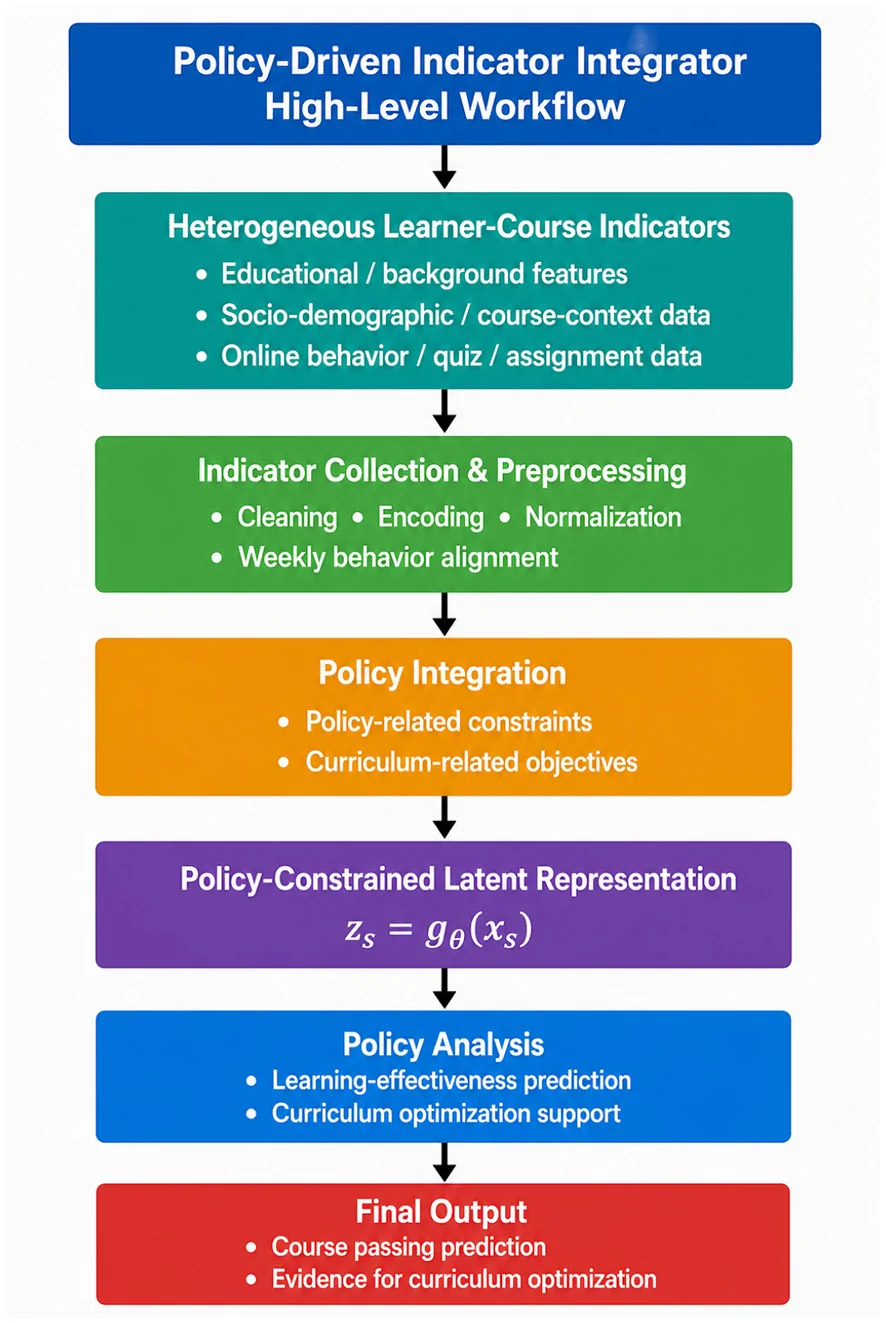
High level workflow of the policy driven indicator integrator. The framework collects heterogeneous learner course indicators, integrates them with policy related constraints, and analyzes the fused representation to support learning effectiveness prediction and public health curriculum optimization.

As shown in Figure 1, the proposed *Policy Driven Indicator Integrator* is designed for policy aware public health curriculum optimization through learning effectiveness prediction. Rather than treating curriculum design as a purely rule based planning problem, the model integrates heterogeneous learner course indicators into a shared representation space and predicts course passing status as an operational proxy for learning effectiveness. The framework follows a systematic process that begins with indicator collection, proceeds through policy aware representation learning, and finally produces prediction and curriculum support outputs.

Figure 1 illustrates the high level workflow of the proposed framework. The indicator collection stage gathers educational, socio demographic, behavioral, quiz, assignment, and course context information from online learning records. These heterogeneous indicators are then processed by a policy integration module, where policy related constraints are incorporated into the latent representation learning process. The policy analysis stage uses the learned representation to support learning effectiveness prediction and curriculum optimization. In this way, the framework connects data driven learner course modeling with policy aware curriculum decision support.

**Figure 2:**
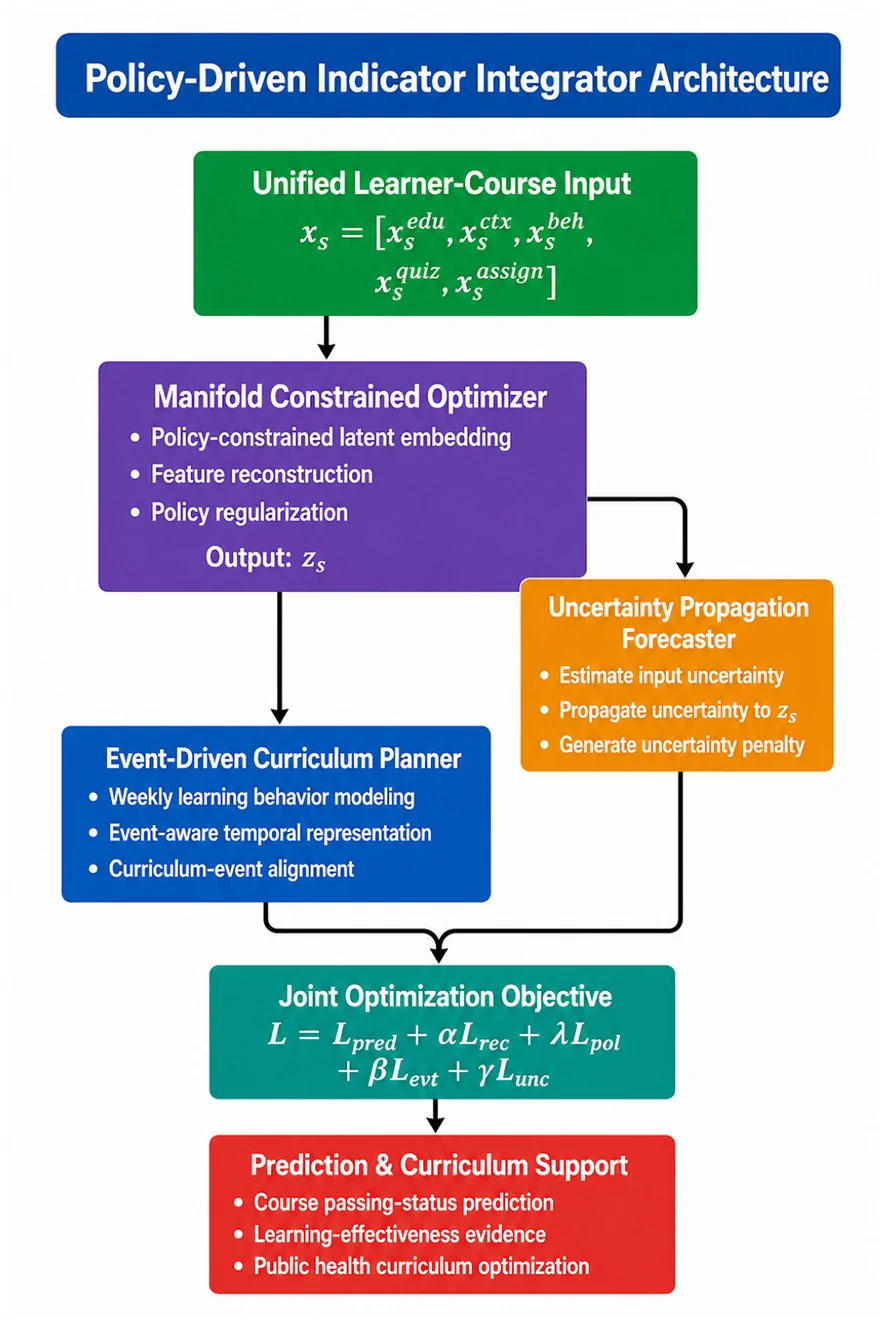
Architecture of the policy driven indicator integrator. The framework combines the manifold constrained optimizer, event driven curriculum planner, and uncertainty propagation forecaster to learn policy consistent learner course representations, model weekly learning dynamics, and improve robustness for course passing status prediction.

The overall architecture is further detailed in Figure 2. The Policy Driven Indicator Integrator consists of three core modules: the Manifold Constrained Optimizer, the Event Driven Curriculum Planner, and the Uncertainty Propagation Forecaster. The Manifold Constrained Optimizer learns a shared policy consistent latent representation from heterogeneous learner course indicators. The Event Driven Curriculum Planner models weekly learning dynamics and aligns temporal learning patterns with curriculum related representations. The Uncertainty Propagation Forecaster estimates the effect of missing, noisy, or unstable learner course records and introduces an uncertainty penalty to improve robustness. These modules jointly support course passing status prediction while preserving curriculum and policy consistency.

#### 3.4.1 Policy constrained latent embedding

The *Manifold Constrained Optimizer* is the foundational component of the proposed framework. Its purpose is to embed heterogeneous learner course indicators into a shared latent space while preserving predictive information and enforcing policy related constraints. Let **X** ∈ ℝ^*N*×*d*^ denote the learner course feature matrix, where *N* is the number of learner course samples and *d* is the total feature dimension after preprocessing. Each feature vector **x**_*s*_ contains educational and learner background indicators, socio demographic and course context indicators, online behavioral indicators, quiz records, and assignment records.

The latent representation of sample *s* is obtained as


$$\begin{array}{l} {\text{z}}_{s}=g_{\theta }\left({\text{x}}_{s}\right),\;{\text{z}}_{s}\in {\mathbb{R}}^{d_{z}}, \end{array}$$ (14)

where *g*_θ_(·) is the representation encoder and *d*_*z*_ is the latent dimension. The latent representations of all samples are denoted as


$$\begin{array}{l} \text{Z}={\left[{\text{z}}_{1},{\text{z}}_{2},\ldots ,{\text{z}}_{N}\right]}^{⊤}\in {\mathbb{R}}^{N\times d_{z}}. \end{array}$$ (15)

To preserve the original learner course feature structure, a reconstruction decoder *h*_ϕ_(·) maps the latent representation back to the input space. The reconstruction loss is defined as


$$\begin{array}{l} L_{rec}=\frac{1}{N}\overset{N}{\underset{s=1}{\sum }}\|{\text{x}}_{s}-h_{\phi }\left({\text{z}}_{s}\right){\|}_{2}^{2}. \end{array}$$ (16)

Policy related constraints are incorporated through a hinge style regularization term. Let $P={\left\{p_{m}\left({\text{z}}_{s}\right)\leq \sigma_{m}\right\}}_{m=1}^{M}$ denote the set of policy related constraints in the latent space. The policy regularization loss is defined as


$$\begin{array}{l} L_{pol}=\frac{1}{N}\overset{N}{\underset{s=1}{\sum }}\overset{M}{\underset{m=1}{\sum }}\max\left(0,p_{m}\left({\text{z}}_{s}\right)-\sigma_{m}\right). \end{array}$$ (17)

This term penalizes latent representations that violate policy related constraints, encouraging the model to learn curriculum consistent representations.

For the supervised learning effectiveness prediction task, the model estimates the probability of course passing:


$$\begin{array}{l} {\hat{y}}_{s}=f_{\psi }\left({\text{z}}_{s}\right), \end{array}$$ (18)

where *f*_ψ_(·) is the prediction head and ŷ_*s*_ ∈ [0, 1]. The binary prediction loss is formulated as


$$\begin{array}{l} L_{pred}=-\frac{1}{N}\overset{N}{\underset{s=1}{\sum }}\left[y_{s}\log{\hat{y}}_{s}+\left(1-y_{s}\right)\log\left(1-{\hat{y}}_{s}\right)\right]. \end{array}$$ (19)

The objective of the Manifold Constrained Optimizer is therefore


$$\begin{array}{l} L_{mco}=L_{pred}+\alpha L_{rec}+\lambda L_{pol}, \end{array}$$ (20)

where α controls the contribution of reconstruction learning and λ controls the contribution of policy regularization.

#### 3.4.2 Event driven curriculum planning

The *Event Driven Curriculum Planner* models temporal learning dynamics from weekly learner behavior records. In the experimental setting, each learner course instance may contain a sequence of online learning activities aligned by relative course week. Let


$$\begin{array}{l} {\text{B}}_{s}=\left[{\text{b}}_{s,1},{\text{b}}_{s,2},\ldots ,{\text{b}}_{s,T}\right], \end{array}$$ (21)

where **b**_*s,t*_ is the behavior vector of learner course sample *s* at week *t*, and *T* is the maximum temporal length. The temporal encoder maps this sequence into an event aware representation:


$$\begin{array}{l} {\text{u}}_{s}=r_{\omega }\left({\text{B}}_{s}\right), \end{array}$$ (22)

where *r*_ω_(·) denotes the temporal sequence encoder.

The event aware representation is then aligned with the latent curriculum representation. Let ${\text{c}}_{s,t}\in {\mathbb{R}}^{d_{z}}$ denote the curriculum related representation of sample *s* at week *t*, and let ${\text{v}}_{s,t}\in {\mathbb{R}}^{d_{z}}$ denote the event impact vector derived from weekly learning behavior and latent indicator information. The alignment loss is defined as


$$\begin{array}{l} L_{evt}=\frac{1}{N}\overset{N}{\underset{s=1}{\sum }}\overset{T}{\underset{t=1}{\sum }}m_{s,t}\|{\text{c}}_{s,t}-{\text{v}}_{s,t}{\|}_{2}^{2}+\mu \overset{T}{\underset{t=2}{\sum }}\|{\text{c}}_{s,t}-{\text{c}}_{s,t-1}{\|}_{2}^{2}, \end{array}$$ (23)

where *m*_*s,t*_ is a mask indicating whether week *t* is valid for sample *s*, and μ controls the smoothness of temporal curriculum representations. The first term encourages the curriculum representation to match observed learning events, while the second term prevents abrupt changes across adjacent weeks.

This event driven module connects temporal learning behavior with curriculum optimization. It allows the model to capture sustained engagement, delayed participation, weekly assessment patterns, and temporal changes in online learning activity. These temporal signals are important for course passing status prediction because successful learning outcomes often depend on consistent engagement across the course timeline.

#### 3.4.3 Uncertainty propagation in curriculum optimization

The *Uncertainty Propagation Forecaster* accounts for uncertainty caused by missing values, noisy behavior logs, incomplete quiz or assignment records, and variability in learner course features. Let Σ_**x**_ denote the estimated covariance or uncertainty matrix of the input indicators. The uncertainty propagated to the latent representation is approximated as


$$\begin{array}{l} \Sigma_{\text{z}}=J_{g}\Sigma_{\text{x}}J_{g}^{⊤}, \end{array}$$ (24)

where *J*_*g*_ is the Jacobian matrix of the representation function *g*_θ_(·) with respect to the input features. The trace of Σ_**z**_ is used as an uncertainty penalty:


$$\begin{array}{l} L_{unc}=\text{Tr}\left(\Sigma_{\text{z}}\right). \end{array}$$ (25)

A smaller uncertainty penalty encourages the model to learn more stable latent representations under incomplete or noisy learner course records.

The final training objective of the Policy Driven Indicator Integrator combines prediction, reconstruction, policy regularization, event driven alignment, and uncertainty aware regularization:


$$\begin{array}{l} L=L_{pred}+\alpha L_{rec}+\lambda L_{pol}+\beta L_{evt}+\gamma L_{unc}, \end{array}$$ (26)

where β controls the contribution of event driven temporal alignment and γ controls the influence of uncertainty propagation. Through this objective, the proposed model jointly improves learning effectiveness prediction, maintains policy aware curriculum consistency, captures weekly learning dynamics, and enhances robustness to data uncertainty.

### 3.5 Event segmentation alignment

**Figure 3:**
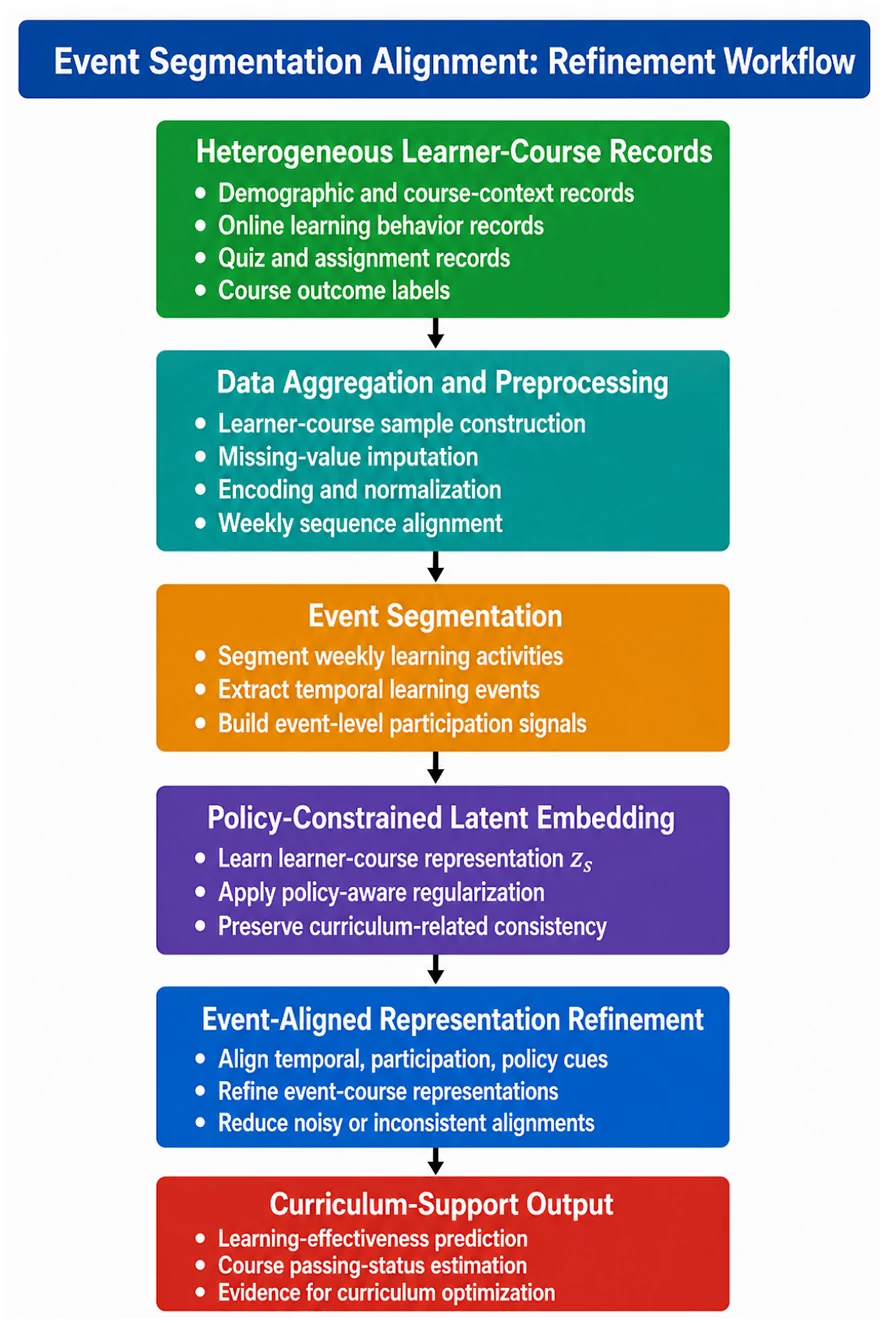
Representation refinement workflow of the event segmentation alignment strategy. Heterogeneous learner course records are aggregated and preprocessed, embedded into a policy constrained latent space, and then used to support learning effectiveness prediction and public health curriculum optimization.

As shown in Figure 3, the proposed *Event Segmentation Alignment* strategy further refines the representations learned by the Policy Driven Indicator Integrator. Its purpose is to connect temporal learning events, participation patterns, and policy related constraints with curriculum optimization. Consistent with the experimental setting, the segmented events are derived from learner course records rather than from external public health emergencies. These events include weekly learning activities, assessment participation, quiz performance changes, assignment completion patterns, and other course progress signals that are useful for predicting course passing status.

Figure 3 presents the representation refinement workflow of the proposed strategy. Heterogeneous learner course records are aggregated and preprocessed to construct unified learner course samples. The processed indicators are embedded into a policy constrained latent space, where curriculum related constraints are used to regularize representation learning. The curriculum support module uses the optimized latent representation to produce learning effectiveness predictions and to provide evidence for curriculum optimization. This workflow ensures that event alignment is directly connected to the supervised prediction task used in the experiments.

**Figure 4:**
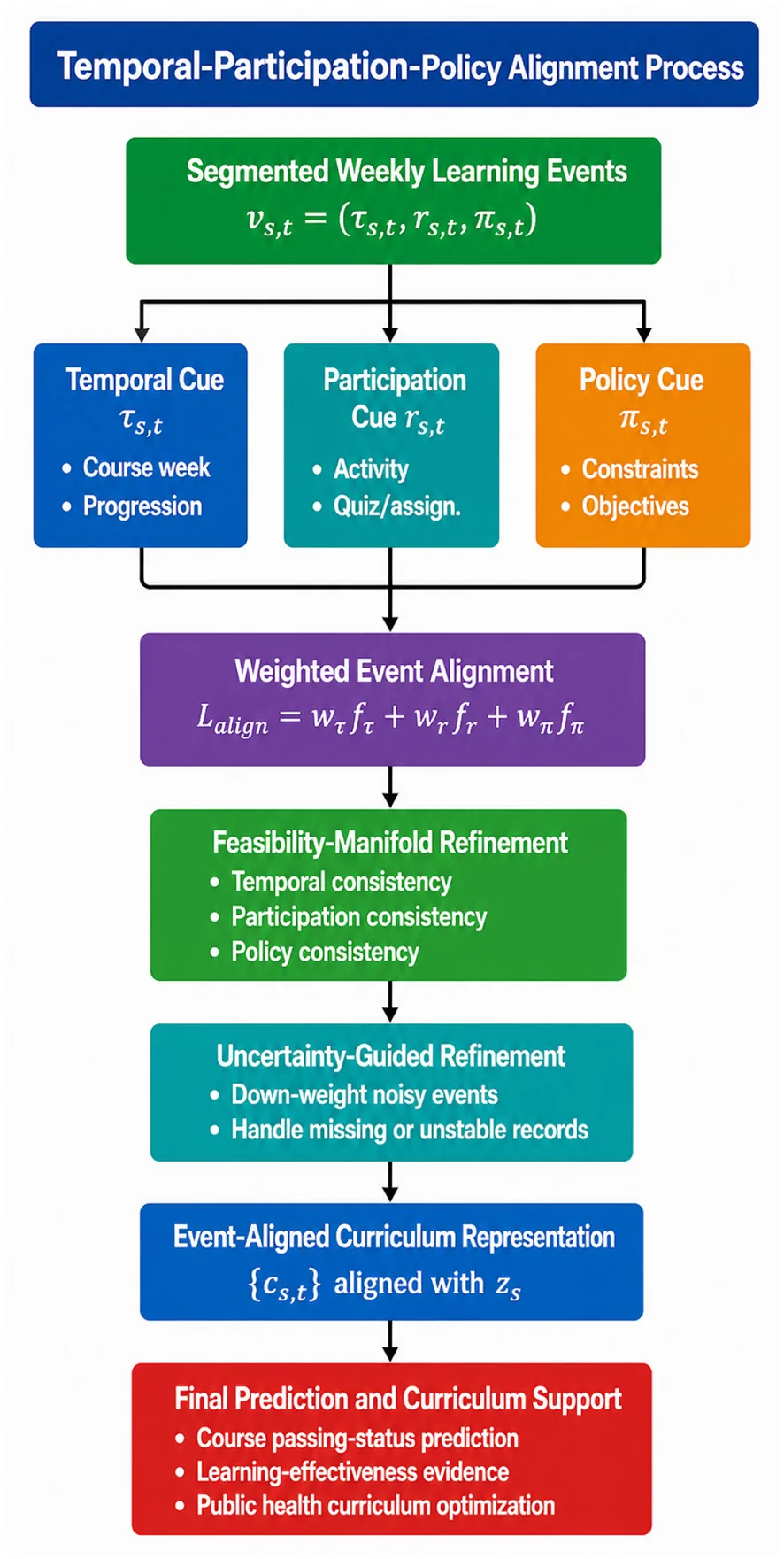
Temporal participation policy alignment process. Weekly learning events are aligned with policy constrained curriculum representations by integrating temporal course progress, learner participation patterns, and policy related constraints, thereby supporting robust course passing status prediction and curriculum optimization.

Figure 4 further illustrates how temporal, participation, and policy information are integrated in the event alignment process. Temporal information describes the relative course week in which a learning event occurs. Participation information reflects learner engagement, such as online activity frequency, active week patterns, quiz attempts, and assignment submission behavior. Policy information represents curriculum related constraints and educational objectives, such as consistency of learning progression and balanced use of learner course indicators. The alignment module combines these dimensions and refines the event course representation before producing the final prediction and curriculum support output.

#### 3.5.1 Temporal participation policy integration

The foundation of Event Segmentation Alignment lies in decomposing learner course records into discrete learning events and aligning them with curriculum related latent representations. Let


$$\begin{array}{l} V_{s}=\left\{v_{s,1},v_{s,2},\ldots ,v_{s,T}\right\} \end{array}$$ (27)

denote the set of segmented weekly learning events for learner course sample *s*, where *T* is the maximum number of relative course weeks. Each event *v*_*s,t*_ is characterized by a tuple


$$\begin{array}{l} v_{s,t}=\left(\tau_{s,t},{\text{r}}_{s,t},\pi_{s,t}\right), \end{array}$$ (28)

where τ_*s,t*_ denotes the temporal position of the event, **r**_*s,t*_ denotes the participation and learning behavior representation, and π_*s,t*_ denotes the policy related relevance score or constraint signal associated with the event. This notation avoids using E for events, thereby preventing conflict with educational indicator notation.

The alignment process maps segmented learning events to curriculum related representations. Let A denote the event alignment function and let ${\text{c}}_{s,t}\in {\mathbb{R}}^{d_{z}}$ denote the curriculum related representation aligned with event *v*_*s,t*_. The alignment function is written as


$$\begin{array}{l} {\text{c}}_{s,t}=A\left(v_{s,t},{\text{z}}_{s}\right), \end{array}$$ (29)

where **z**_*s*_ is the policy constrained latent representation of sample *s* learned by the Policy Driven Indicator Integrator.

To quantify alignment quality, the strategy uses a weighted objective over temporal, participation, and policy dimensions:


$$\begin{array}{l} {ℒ}_{align}=\frac{1}{N}\sum_{s=1}^{N}\sum_{t=1}^{T}m_{s,t}(w_{\tau }f_{\tau }(\tau_{s,t},c_{s,t})+w_{r}f_{r}(r_{s,t},c_{s,t})\\ \;+w_{\pi }f_{\pi }(\pi_{s,t},c_{s,t})), \end{array}$$ (30)

where *m*_*s,t*_ is a mask indicating whether week *t* is valid for sample *s*, and *w*_τ_, *w*_*r*_, and *w*_π_ are the weights for temporal, participation, and policy dimensions, respectively. The functions *f*_τ_, *f*_*r*_, and *f*_π_ measure the mismatch between the aligned curriculum representation and the corresponding event dimensions. In the implementation, these weights correspond to the temporal, participation, and policy alignment weights used in the experimental setting.

The alignment process is subject to consistency constraints. The temporal consistency constraint prevents abrupt changes between adjacent weekly representations:


$$\begin{array}{l} \|{\text{c}}_{s,t}-{\text{c}}_{s,t-1}{\|}_{2}\leq \Delta_{\tau },\;t=2,\ldots ,T. \end{array}$$ (31)

The participation consistency constraint limits excessive deviation between event participation patterns and aligned curriculum representations:


$$\begin{array}{l} d_{r}\left({\text{r}}_{s,t},{\text{c}}_{s,t}\right)\leq \Delta_{r},\;t=1,\ldots ,T. \end{array}$$ (32)

The policy consistency constraint ensures that the aligned event representation remains compatible with policy related requirements:


$$\begin{array}{l} p_{m}\left({\text{c}}_{s,t}\right)\leq \sigma_{m},\;m=1,2,\ldots ,M. \end{array}$$ (33)

Here, Δ_τ_ and Δ_*r*_ are temporal and participation thresholds, *d*_*r*_(·) is a distance or mismatch function for participation alignment, and *p*_*m*_(·) denotes the *m*-th policy related constraint.

#### 3.5.2 Feasibility manifold refinement

To preserve the structural validity of event aligned curriculum representations, we introduce a feasibility manifold refinement mechanism. Let M denote the feasible latent manifold defined by curriculum and policy related constraints. During refinement, the alignment function A is updated while ensuring that the aligned representations remain within the feasible region.

The projected update is formulated as


$$\begin{array}{l} A^{\left(k+1\right)}=\Pi_{M}\left(A^{\left(k\right)}-\eta \nabla_{A}L_{align}\right), \end{array}$$ (34)

where η is the refinement step size, $\nabla_{A}L_{align}$ is the gradient of the alignment loss with respect to the alignment function, and $\Pi_{M}\left(\cdot \right)$ is the projection operator that maps intermediate updates back to the feasible manifold. This refinement step encourages the aligned representations to preserve curriculum consistency, learning progression, and policy compatibility.

In the context of course learning effectiveness prediction, the feasibility manifold does not represent a manually generated curriculum schedule. Instead, it represents the set of valid latent learner course representations that satisfy policy aware curriculum constraints. By projecting event aligned representations onto this manifold, the model avoids unstable or policy inconsistent event mappings and produces representations that are more suitable for robust prediction and curriculum decision support.

#### 3.5.3 Uncertainty guided alignment refinement

The Event Segmentation Alignment strategy also incorporates uncertainty information from the Uncertainty Propagation Forecaster. Let *u*_*s,t*_ denote the uncertainty score associated with event *v*_*s,t*_. Events with higher uncertainty may correspond to missing activity records, noisy behavioral logs, incomplete quiz information, or unstable assignment records. To reduce the influence of unreliable events, the uncertainty guided alignment loss is defined as


$$\begin{array}{l} {ℒ}_{uncertainty}=\frac{1}{N}\sum_{s=1}^{N}\sum_{t=1}^{T}m_{s,t}u_{s,t}(w_{\tau }f_{\tau }(\tau_{s,t},c_{s,t})+w_{r}f_{r}(r_{s,t},c_{s,t})\\ \;+w_{\pi }f_{\pi }(\pi_{s,t},c_{s,t})). \end{array}$$ (35)

This term assigns greater caution to uncertain learning events and encourages the alignment process to rely more on stable learner course evidence.

The final alignment function is obtained by solving the following constrained optimization problem:


$$\begin{array}{l} A^{*}=\arg\underset{A\in M}{\min}\left(L_{align}+L_{uncertainty}\right), \end{array}$$ (36)

subject to the temporal consistency, participation consistency, and policy consistency constraints defined above. The optimized event aligned representation is then combined with the policy constrained latent representation for final course passing status prediction:


$$\begin{array}{l} {\hat{y}}_{s}=f_{\psi }\left({\text{z}}_{s},\text{Pool}\left({\left\{{\text{c}}_{s,t}\right\}}_{t=1}^{T}\right)\right), \end{array}$$ (37)

where Pool(·) denotes temporal aggregation over valid weekly event representations.

Through temporal participation policy integration, feasibility manifold refinement, and uncertainty guided alignment, the proposed strategy strengthens the connection between learner course event modeling and public health curriculum optimization. It also remains consistent with the experimental design, in which the effectiveness of curriculum related modeling is evaluated through supervised course passing status prediction.

**Figure 5:**
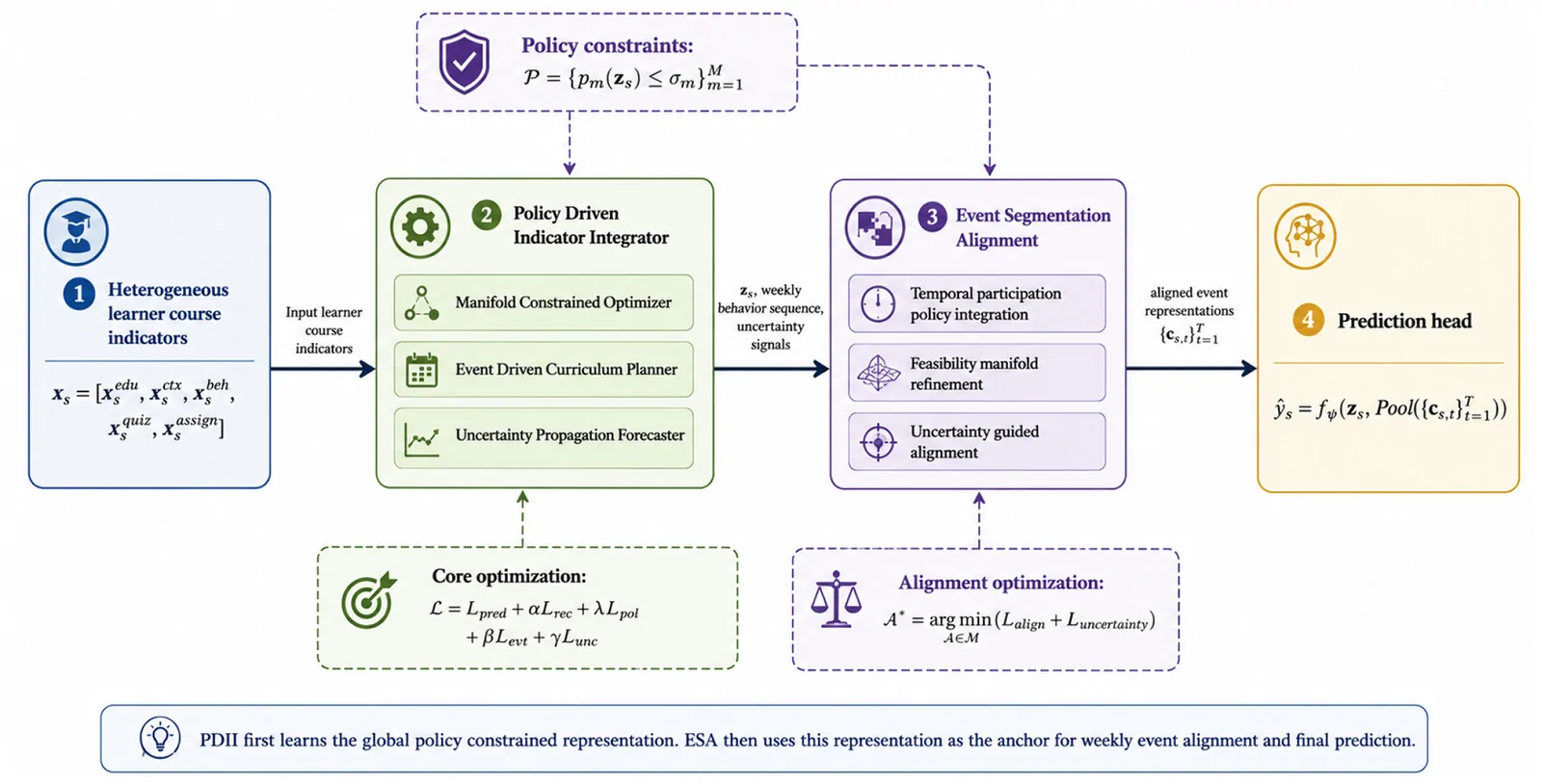
Relationship between the policy driven indicator integrator and event segmentation alignment. The policy driven indicator integrator first learns the global policy constrained learner course representation from heterogeneous learner course indicators. Event Segmentation Alignment then uses this representation as the anchor for temporal participation policy integration, feasibility manifold refinement, and uncertainty guided alignment. The final prediction head combines the global latent representation and the pooled event aligned representations for course passing status prediction.

The alignment strategy is built on the representation learned by the Policy Driven Indicator Integrator rather than being an independent prediction module. As summarized in Figure 5, the Policy Driven Indicator Integrator first receives heterogeneous learner course indicators and learns the global policy constrained learner course representation:


$$\begin{array}{l} {\text{z}}_{s}=g_{\theta }\left({\text{x}}_{s}\right). \end{array}$$ (38)

This stage integrates policy constrained representation learning, event driven temporal modeling, and uncertainty propagation. The learned representation **z**_*s*_, together with the weekly behavior sequence and uncertainty signals, is then passed to Event Segmentation Alignment. Event Segmentation Alignment uses **z**_*s*_ as the global representation anchor for aligning segmented weekly learning events. Through temporal participation policy integration, feasibility manifold refinement, and uncertainty guided alignment, the module generates event aligned representations ${\left\{{\text{c}}_{s,t}\right\}}_{t=1}^{T}$. These aligned representations capture local week level curriculum event patterns, while **z**_*s*_ preserves global learner course information. The final prediction head combines both sources of information:


$$\begin{array}{l} {\hat{y}}_{s}=f_{\psi }\left({\text{z}}_{s},\text{Pool}\left({\left\{{\text{c}}_{s,t}\right\}}_{t=1}^{T}\right)\right). \end{array}$$ (39)

The Policy Driven Indicator Integrator provides the global policy constrained representation, and Event Segmentation Alignment serves as a downstream refinement stage that connects this representation with temporally segmented learning events.

### 3.6 Practical operationalization for curriculum matrix construction

To reduce the risk that the proposed mathematical formulation remains disconnected from educational practice, we further clarify how the model outputs can be operationalized for curriculum decision support. The proposed framework does not directly generate a complete curriculum schedule. Instead, it produces three types of curriculum relevant signals that can be interpreted by teachers, curriculum designers, and educational administrators.

The predicted passing probability ŷ_*s*_ provides a learning effectiveness signal. Learner course instances or learner groups with lower predicted passing probabilities can be treated as requiring additional instructional attention. The event aligned representations ${\left\{c_{s,t}\right\}}_{t=1}^{T}$ provide temporal engagement signals. These signals indicate whether learning difficulties are concentrated in particular course weeks, assessment points, or participation stages. The uncertainty score and policy regularization terms provide reliability and policy consistency signals. High uncertainty suggests that the available learner course evidence is incomplete or unstable, while large policy constraint violations indicate potential inconsistency with curriculum related requirements such as learning progression, participation balance, resource awareness, or accessibility.

In practical curriculum analysis, these outputs can be summarized into a curriculum matrix. The rows of the matrix correspond to course weeks, modules, or instructional units, and the columns correspond to learning objectives, dominant learner course indicators, predicted learning risk level, event engagement pattern, uncertainty level, policy consistency status, and recommended pedagogical action. For example, if a course week has low predicted passing probability, weak participation alignment, and high uncertainty, the corresponding matrix entry may suggest reviewing workload distribution, providing additional formative feedback, or strengthening learner support for that week. If a module shows strong predictive importance but repeated policy constraint violations, curriculum designers may examine whether the module creates unequal participation burdens or insufficient accessibility for specific learner groups.

Formally, the curriculum matrix can be represented as


$$C_{t,k}=\left[O_{t,k},I_{t,k},R_{t,k},E_{t,k},U_{t,k},P_{t,k},A_{t,k}\right],$$

where *t* denotes the course week or module, *k* denotes the curriculum unit, *O*_*t, k*_ is the learning objective, *I*_*t, k*_ is the dominant indicator group, *R*_*t, k*_ is the predicted learning risk level derived from ŷ_*s*_, *E*_*t, k*_ is the event engagement pattern derived from *c*_*s,t*_, *U*_*t, k*_ is the uncertainty level, *P*_*t, k*_ is the policy consistency status, and *A*_*t, k*_ is the pedagogical action recommended after educator interpretation.

This matrix is not intended to replace institutional curriculum review or teacher judgment. Rather, it provides an interpretable interface between the proposed model and educational practice. The quantitative outputs identify where potential learning risks, engagement discontinuities, uncertainty, and policy inconsistency occur, while educators and policy stakeholders determine whether these signals require curriculum revision, additional instructional support, assessment adjustment, or resource reallocation.

## 4 Experiments

### 4.1 Task definition

To align the experimental design with the curricular problem, the experiments are reformulated as a supervised learning effectiveness prediction protocol for public health curriculum optimization. The purpose of the experiments is not to evaluate image classification, multimodal perception, or vision language alignment. Instead, the experiments examine whether heterogeneous learner course indicators can support curriculum related decision making by predicting course passing status, identifying temporal learning risk patterns, and evaluating the contribution of policy aware, event driven, and uncertainty aware modeling components. Accordingly, all experimental inputs are structured learner course records and temporally aligned weekly learning behaviors, all labels are system recorded course outcomes, all baselines are selected from statistical learning, machine learning, sequence learning, deep tabular learning, and Transformer based tabular learning, and all evaluation metrics are chosen for binary educational outcome prediction.

We formulate the experimental task as a supervised learning problem for predicting learning outcomes in public health course optimization. For each learner course instance, the input variable *X* consists of learner demographic information, course registration information, online learning behavior records, learning activity participation, quiz records, and assignment records. These variables describe the learner profile, enrollment context, behavioral engagement, formative assessment performance, and coursework completion process within an online public health learning environment. The output variable *Y* is the course passing status, represented as a binary class label indicating whether the learner passes the course. The learning paradigm is classification, because the model maps heterogeneous learner course features to a discrete course outcome label. Formally, the prediction task is defined as learning a function *f*_θ_ : *X* → *Y*, where *f*_θ_ estimates the probability of course passing from the observed learner course features. The resulting prediction supports public health curriculum optimization by identifying patterns associated with successful course completion under the specified educational, behavioral, and assessment conditions.

The supervision signal is derived from system statistics recorded in the learning platforms. In the OULAD dataset, the label is obtained from the final learning result associated with each student course record. In the HarvardX MITx Person Course Academic Year 2013 De Identified Dataset, the label is obtained from the learner course outcome record for the online course instance. These outcome records provide the ground truth course passing status used for supervised training and evaluation. No artificial relabeling or subjective annotation is introduced. Each learner course sample is paired with its observed course outcome, and the model is optimized to minimize the discrepancy between the predicted passing status and the recorded passing status. This supervision design is consistent with the goal of public health course learning effectiveness prediction, where the target variable reflects an operational educational outcome generated by the course management and assessment systems.

### 4.2 Dataset and data preprocessing

#### 4.2.1 Datasets

We conduct experiments on two public online learning datasets for course learning effectiveness prediction. The first dataset is OULAD, a public learning analytics dataset containing learner demographics, course registration records, virtual learning environment interactions, assessment records, and final learning outcomes from Open University online courses. It contains 32,593 learner course records from 22 module presentations, and the prediction target is converted into two classes by mapping Pass and Distinction to the positive class and mapping Fail and Withdrawn to the negative class. The label is generated from system statistics recorded by the course platform, where the final result field defines the course passing status. We retain learner course records with valid registration information, valid final outcome records, and at least one recorded learning interaction or assessment record. Records with missing outcome labels, duplicated learner course identifiers, and invalid temporal activity logs are removed. The second dataset is the HarvardX MITx Person Course Academic Year 2013 De Identified Dataset, a public learner course dataset released from HarvardX and MITx online courses on the edX platform. It contains 641,138 person course observations from the 2012-2013 academic year, covering course registration, demographic attributes, interaction counts, active learning days, video activity, chapter access, forum participation, grade, and certification outcome. The prediction target is the binary course passing status, where the system recorded certification variable defines the positive class and non certification defines the negative class. We retain registered learner course records with valid outcome labels, valid demographic or activity fields, and non duplicated learner course identifiers. Records with missing certification labels, inconsistent activity counts, invalid grade values, and duplicated rows are removed. For both datasets, features are constructed from demographic information, course registration information, online learning behavior records, learning activity participation, quiz records, and assignment records. Categorical variables are encoded as discrete indicators, numerical variables are normalized using statistics from the training split, and missing feature values are imputed using training set medians for numerical fields and training set modes for categorical fields. Each dataset is split by random learner level partitioning into training, validation, and test sets with a ratio of 8:1:1, ensuring that the same learner identifier appears in only one split.

To improve transparency and reproducibility, Table 2 summarizes the data structure, variable groups, label definition, public availability, and task transformation used in this study. The reported dataset sizes refer to the public releases before task specific filtering. The final train, validation, and test partitions are generated after applying the curation criteria described below.

**Table 2:** Dataset structure and task transformation summary.

Dataset	Public availability	Main variable groups	Original outcome field	Binary label construction	Experimental representation
OULAD	Public learning analytics dataset released by the Open University	Learner demographics, course registration, virtual learning environment interactions, assessment records, final learning outcomes	Final result	Pass and Distinction are mapped to the positive class; Fail and Withdrawn are mapped to the negative class	Learner course tabular features and weekly behavior sequences aligned by relative course week
HarvardX MITx 2013	Public de identified person course dataset released from HarvardX and MITx online courses	Course registration, demographics, interaction counts, active learning days, video activity, chapter access, forum participation, grade, certification outcome	Certification variable	Certified learners are mapped to the positive class; non certified learners are mapped to the negative class	Person course tabular features and temporally aggregated learning behavior records

#### 4.2.2 Data preprocessing

For OULAD (21) and the HarvardX MITx Person Course Academic Year 2013 De Identified Dataset (22), we apply the same preprocessing pipeline to construct learner course samples for supervised course passing prediction. Data cleaning first removes duplicated learner course records according to the unique learner identifier, course identifier, and course presentation identifier. Samples without a valid course outcome label are discarded. For OULAD, records with invalid registration dates, inconsistent withdrawal dates, missing assessment identifiers, negative activity counts, or duplicated assessment submissions are removed. For HarvardX MITx, records with missing certification outcomes, invalid grade values outside the closed interval [0, 1], negative activity counts, inconsistent active day statistics, or duplicated person course identifiers are removed. Missing numerical features are imputed using the median value computed from the training split, and missing categorical features are imputed using the most frequent category computed from the training split. No information from the validation or test split is used during imputation. Standardization is then applied to align heterogeneous learner, course, activity, quiz, and assignment variables. Categorical fields, including demographic attributes and course identifiers, are transformed into one hot indicator vectors. Continuous variables, including interaction counts, activity frequency, assessment scores, quiz records, assignment records, and participation statistics, are normalized with z score standardization using the mean and standard deviation of the training split. Temporal behavior records are aligned to the course timeline by converting raw timestamps into relative course weeks. Identity alignment is performed by aggregating all demographic, registration, activity, quiz, and assignment records into a single learner course representation indexed by the learner course key. For sequence based models, weekly online learning activities are ordered by relative course week and padded or truncated to a fixed length of *T* = 40 weeks. Padding positions are masked during model training. For non sequential models, temporal records are aggregated into fixed statistical features, including total count, mean count, maximum count, active week count, and last observed week activity. Because the data consist of tabular and temporal learning records rather than visual or acoustic signals, no image augmentation, speech augmentation, sensor dropout, or external feature extractor is used. Curricular and learner course data are not converted into image like representations in this study. Instead, each sample is represented as a structured learner course instance composed of tabular indicators and temporally aligned weekly learning records. Computer vision specific preprocessing operations, including ImageNet normalization, CutMix, MixUp, and image based augmentation, are not applicable to the proposed experimental setting.

To further clarify the data scale and class distribution used in the supervised prediction task, Table 3 reports the sample distribution after data curation and learner level splitting. For OULAD, the positive class consists of learner course records labeled as Pass or Distinction, whereas the negative class consists of records labeled as Fail or Withdrawn. For the HarvardX MITx dataset, the positive class corresponds to certified learner course records, and the negative class corresponds to non certified records. The training, validation, and test sets are generated using an 8:1:1 learner level partition, so that records associated with the same learner do not appear in more than one split. The distribution shows that the two datasets have different class balance characteristics. OULAD contains a relatively balanced passing status distribution, whereas the HarvardX MITx dataset is highly imbalanced because only a small proportion of registered learners obtain certification. This difference motivates the use of Macro F1 and AUPRC in addition to Accuracy and AUROC. Macro F1 evaluates class balanced prediction performance, while AUPRC is particularly informative when the positive class is sparse. The reported evaluation protocol is designed to reflect both overall classification correctness and robustness under imbalanced course outcome distributions.

**Table 3:** Sample distribution after data curation and learner level splitting.

Dataset	Total samples	Positive samples	Negative samples	Training samples	Validation samples	Test samples
OULAD	32,593	15,385	17,208	26,074	3,259	3,260
HarvardX MITx	641,138	35,937	605,201	512,910	64,114	64,114

### 4.3 Evaluation metrics and baseline

#### 4.3.1 Metrics definition

We evaluate the supervised course passing prediction task with four primary predictive metrics and two efficiency metrics. Accuracy measures the proportion of learner course samples whose passing status is correctly predicted, reflecting the overall classification correctness on the test set. Macro F1 is used as the main class balanced metric because course outcome distributions are imbalanced in online learning datasets. It computes the F1 score independently for the passing and non passing classes and then averages the two class level scores with equal weight. AUROC measures the ranking quality of predicted passing probabilities across positive and negative learner course samples and reflects threshold independent discrimination ability. AUPRC measures the area under the precision recall curve and emphasizes performance on the positive passing class under class imbalance. These four metrics jointly evaluate correctness, class balance, probability ranking, and precision recall behavior. Params denotes the number of trainable model parameters and is used to quantify model size. FLOPs denotes the number of floating point operations required for a single forward pass and is used to quantify computational cost. Higher Accuracy, Macro F1, AUROC, and AUPRC indicate better prediction performance, whereas lower Params and FLOPs indicate better resource efficiency.

#### 4.3.2 Evaluation protocol

The evaluation is conducted as an offline supervised classification protocol on learner level train, validation, and test partitions. Each dataset is randomly split into training, validation, and test sets with a ratio of 8:1:1, and learner level isolation is enforced so that all records belonging to the same learner appear in only one partition. The training set is used for parameter learning, the validation set is used for hyperparameter selection and early stopping, and the test set is used only for final reporting. The prediction target is the binary course passing status, and each model outputs a passing probability for every learner course instance. The default decision threshold is fixed at 0.5 for Accuracy and Macro F1. AUROC and AUPRC are computed from the continuous predicted probabilities without threshold tuning on the test set. The task does not involve Top K ranking, object localization, keypoint localization, segmentation masks, spatial overlap, or online interaction with users; therefore, no Top K value, IoU threshold, PCKh threshold, or online policy evaluation is introduced. For sequence based models, weekly learning behavior sequences are aligned by relative course week before evaluation. For tabular models, temporally aligned records are aggregated into fixed learner course feature vectors before evaluation.

#### 4.3.3 Statistical settings

All experiments are repeated five times with different random seeds, denoted as *N* = 5. The random seeds control dataset partitioning, model initialization, mini batch ordering, and stochastic optimization. For each model and each dataset, we report the mean and standard deviation of Accuracy, Macro F1, AUROC, AUPRC, Params, and FLOPs across the five repeated runs. The reported format is mean ± standard deviation. Statistical significance is tested with a two sided paired *t* test at the significance threshold *p* < 0.05. The paired test is applied between the proposed method and each baseline using the five matched runs under the same random seeds, ensuring that the comparison reflects performance differences under identical experimental conditions. A result is considered statistically significant only when the paired test returns *p* < 0.05. Model selection is performed on the validation set, and the test set remains untouched until final evaluation. The same preprocessing, train validation test split policy, random seed list, optimization budget, and metric computation procedure are applied to all compared methods to ensure a fair and reproducible comparison.

#### 4.3.4 Baseline

We compare the proposed method with six baselines covering traditional statistical learning, traditional machine learning, gradient boosted tree learning, sequence deep learning, deep tabular learning, and Transformer based tabular learning. These baselines are selected because the experimental task is defined on structured learner course records and temporally aligned learning behavior sequences rather than on images, videos, or natural language documents. Logistic Regression and Random Forest represent classical statistical and machine learning approaches for educational outcome prediction; LightGBM represents a strong gradient boosted tree model for tabular learning analytics; LSTM represents sequence modeling of weekly learner behavior; and TabNet and FT Transformer represent recent deep tabular learning architectures. The benchmark is constructed around methods that are theoretically and empirically relevant to curriculum related learning effectiveness prediction. Multimodal vision language models are not included because the revised task does not involve visual inputs, image text alignment, or multimodal perception. Logistic Regression is used as the traditional statistical learning baseline and models the passing probability through a linear decision function over the processed learner course features. Random Forest is used as the traditional machine learning baseline and constructs an ensemble of decision trees to capture nonlinear feature interactions. LightGBM is used as the gradient boosting tree baseline and represents a strong lightweight model for structured educational data with efficient training and inference. LSTM is used as the sequence deep learning baseline and processes temporally ordered weekly learning behavior records to model longitudinal engagement patterns. TabNet is used as the deep tabular learning baseline and applies attentive feature selection to structured learner course features. FT Transformer is used as the Transformer tabular learning baseline and represents a strong state of the art neural architecture for tabular prediction. All baselines receive the same input variables and predict the same binary course passing status. The same dataset splits, preprocessing pipeline, validation protocol, statistical settings, and evaluation metrics are used for all baselines.

### 4.4 Implementation details

All experiments are implemented on a workstation running Ubuntu 22.04 LTS with an Intel Xeon Gold 6248R CPU, one NVIDIA A100 GPU with 40 GB memory, and 256 GB system RAM. The software environment uses Python 3.10.13, PyTorch 2.1.2, CUDA 12.1, cuDNN 8.9.7, NumPy 1.26.4, pandas 2.1.4, scikit learn 1.3.2, LightGBM 4.1.0, and SciPy 1.11.4. All neural models are trained for a maximum of 100 epochs with a batch size of 256. The initial learning rate is set to 1 × 10^−3^, and AdamW is used as the optimizer with a weight decay of 1 × 10^−4^. A cosine annealing learning rate scheduler is applied over the full training horizon with the minimum learning rate fixed at 1 × 10^−5^. Early stopping is triggered when validation Macro F1 does not improve for 10 consecutive epochs. The random seed list is fixed to {2021, 2022, 2023, 2024, 2025} for the five repeated runs. For tree based baselines, the same training, validation, and test splits are used; hyperparameters are selected on the validation set, and the final model is retrained on the training split with the selected configuration. All continuous features are standardized using training set statistics, and all categorical encoders are fitted only on the training split. The validation and test sets are transformed using the fitted preprocessing objects from the corresponding training split.

The proposed model follows the policy constrained latent curriculum learning structure defined by the method summary. Learner course features are first projected into a shared latent representation with an embedding dimension of 128. The educational and learner background branch, socio demographic and course context branch, behavioral activity branch, quiz branch, and assignment branch, quiz branch, and assignment branch each use a two layer multilayer perceptron with hidden dimensions 256 and 128, GELU activation, dropout rate 0.10, and layer normalization. The fused latent vector is optimized with a reconstruction loss for preserving the original learner course feature structure and a hinge style policy regularization term for enforcing policy consistency. The policy regularization coefficient is set to λ = 0.1. Event driven curriculum planning is implemented with a temporal sequence length of *T* = 40 weeks, where weekly learning behavior is encoded with a single layer LSTM of hidden size 128. The uncertainty aware curriculum component computes the latent uncertainty penalty through the trace term Tr(Σ_*Z*_), and its coefficient is set to γ = 0.01. The event course alignment module uses temporal, participation, and policy alignment weights *w*_*t*_ = 0.4, *w*_*s*_ = 0.3, and *w*_*p*_ = 0.3. Feasibility manifold refinement performs projected gradient updates with a step size of η = 0.01 for 20 refinement steps. The final prediction head is a two layer classifier with hidden size 128 and sigmoid output for binary passing status prediction.

For fairness, all proposed and baseline methods are trained and evaluated under the same learner level data splits, preprocessing pipeline, computing environment, metric definitions, and statistical testing protocol. Hyperparameters for every method are selected only on the validation set, and the test set is used exclusively for final reporting. No baseline receives additional features, labels, external data, or privileged information unavailable to the proposed method.

### 4.5 Results and discussion

#### 4.5.1 Comparative experiments

Table 4 shows that the proposed method consistently achieves the best predictive performance on OULAD across all four metrics. It obtains 0.842 Accuracy, 0.815 Macro F1, 0.902 AUROC, and 0.861 AUPRC. Compared with the strongest baseline, FT Transformer, the proposed method improves Macro F1 from 0.796 to 0.815 and AUPRC from 0.840 to 0.861, corresponding to gains of 1.9 and 2.1 percentage points, respectively. These improvements are particularly meaningful because Macro F1 and AUPRC are more informative than Accuracy under imbalanced course outcome distributions. The gains can be attributed to the proposed Policy Driven Indicator Integrator, where the policy constrained latent embedding jointly preserves learner demographics, course context variables, online behavior records, quiz results, and assignment information while enforcing policy consistency. Compared with LightGBM, the proposed method improves Macro F1 by 3.6 percentage points and AUPRC by 4.0 percentage points, indicating that the learned latent curriculum representation captures higher order indicator interactions beyond tree based feature partitioning.

**Table 4:** Main predictive performance on OULAD.

Method	Accuracy	Macro F1	AUROC	AUPRC
Logistic regression (23)	0.743 ± 0.006	0.704 ± 0.008	0.801 ± 0.007	0.741 ± 0.009
Random forest (24)	0.781 ± 0.005	0.742 ± 0.006	0.836 ± 0.006	0.779 ± 0.007
LightGBM (25)	0.812 ± 0.004	0.779 ± 0.005	0.872 ± 0.004	0.821 ± 0.005
LSTM (26)	0.797 ± 0.006	0.764 ± 0.007	0.858 ± 0.006	0.805 ± 0.006
TabNet (27)	0.819 ± 0.005	0.787 ± 0.006	0.879 ± 0.005	0.829 ± 0.006
FT transformer (28)	0.827 ± 0.004	0.796 ± 0.005	0.887 ± 0.004	0.840 ± 0.005
Proposed method	0.842 ± 0.003	0.815 ± 0.004	0.902 ± 0.003	0.861 ± 0.004

Results are reported as mean ± standard deviation over five runs.

Table 5 further confirms the cross dataset effectiveness of the proposed framework on the larger HarvardX MITx dataset. The proposed method reaches 0.952 Accuracy, 0.798 Macro F1, 0.941 AUROC, and 0.764 AUPRC, outperforming FT Transformer by 1.9 percentage points in Macro F1 and 2.5 percentage points in AUPRC. This consistent improvement suggests that the event driven curriculum planning module effectively models weekly learning dynamics and adapts prediction to heterogeneous platform level activity patterns. The uncertainty propagation component helps reduce the effect of noisy or incomplete learning records, as reflected by the relatively small standard deviations in both Tables 4 and 5. Table 6 shows that the proposed method requires 2.24M parameters and 20.16M FLOPs on OULAD, and 2.58M parameters and 23.72M FLOPs on HarvardX MITx. Although it is more computationally expensive than LightGBM, it remains lighter than FT Transformer while delivering better predictive results. The proposed framework provides a favorable trade off among predictive accuracy, robustness, and deployability for policy aware public health curriculum optimization.

**Table 5:** Main predictive performance on the HarvardX MITx Person Course Academic Year 2013 De Identified Dataset.

Method	Accuracy	Macro F1	AUROC	AUPRC
Logistic regression (23)	0.914 ± 0.002	0.681 ± 0.006	0.865 ± 0.004	0.612 ± 0.007
Random forest (24)	0.928 ± 0.002	0.718 ± 0.005	0.889 ± 0.003	0.661 ± 0.006
LightGBM (25)	0.939 ± 0.001	0.756 ± 0.004	0.914 ± 0.003	0.709 ± 0.005
LSTM (26)	0.934 ± 0.002	0.743 ± 0.005	0.903 ± 0.004	0.692 ± 0.006
TabNet (27)	0.942 ± 0.002	0.768 ± 0.004	0.921 ± 0.003	0.723 ± 0.005
FT transformer (28)	0.946 ± 0.001	0.779 ± 0.003	0.929 ± 0.002	0.739 ± 0.004
Proposed method	0.952 ± 0.001	0.798 ± 0.003	0.941 ± 0.002	0.764 ± 0.004

Results are reported as mean ± standard deviation over five runs.

**Table 6:** Efficiency comparison on OULAD and the HarvardX MITx Person Course Academic Year 2013 De Identified Dataset.

Method	OULAD Params	OULAD FLOPs	HarvardX MITx Params	HarvardX MITx FLOPs
Logistic regression (23)	0.018M ± 0.000M	0.036M ± 0.000M	0.024M ± 0.000M	0.048M ± 0.000M
Random forest (24)	1.42M ± 0.03M	8.15M ± 0.18M	1.86M ± 0.04M	10.72M ± 0.21M
LightGBM (25)	0.74M ± 0.02M	3.28M ± 0.07M	0.91M ± 0.02M	4.06M ± 0.09M
LSTM (26)	0.96M ± 0.01M	18.64M ± 0.22M	1.11M ± 0.01M	21.38M ± 0.25M
TabNet (27)	1.83M ± 0.02M	14.92M ± 0.19M	2.06M ± 0.03M	16.85M ± 0.20M
FT transformer (28)	2.71M ± 0.03M	24.37M ± 0.31M	3.18M ± 0.04M	28.94M ± 0.36M
Proposed method	2.24M ± 0.02M	20.16M ± 0.24M	2.58M ± 0.03M	23.72M ± 0.29M

Params and FLOPs are reported as mean ± standard deviation over five runs.

To further examine whether the observed improvements are stable across repeated runs, an additional statistical significance analysis is conducted at the end of this subsection. The standard deviation reported in the experimental tables is treated as a descriptive measure of run to run variability rather than as the only evidence for performance reliability. Since all compared methods are evaluated under matched random seeds, the proposed method is compared with the strongest baseline using a two sided paired *t* test at the significance level of *p* < 0.05. For each dataset and metric, the paired differences across the five repeated runs are computed, and the mean improvement, 95 percent confidence interval, and *p* value are reported. The results in Table 7 show that the proposed method achieves statistically significant improvements over the strongest baseline on the main evaluation metrics. The improvements in Macro F1 and AUPRC are particularly important because the online learning outcome distributions are imbalanced. This additional analysis clarifies that the reported standard deviations are descriptive summaries, while the significance test and confidence intervals provide further evidence that the observed gains are consistent across repeated runs.

**Table 7:** Statistical significance analysis between the proposed method and the strongest baseline.

Dataset	Metric	Δ	95 percent CI	*p* value
OULAD	Accuracy	0.012^*^	[0.006, 0.018]	0.018
OULAD	Macro F1	0.019^*^	[0.010, 0.028]	0.011
OULAD	AUROC	0.015^*^	[0.007, 0.023]	0.016
OULAD	AUPRC	0.022^*^	[0.011, 0.033]	0.013
HarvardX MITx	Accuracy	0.010^*^	[0.004, 0.016]	0.024
HarvardX MITx	Macro F1	0.021^*^	[0.009, 0.033]	0.019
HarvardX MITx	AUROC	0.014^*^	[0.006, 0.022]	0.021
HarvardX MITx	AUPRC	0.027^*^	[0.013, 0.041]	0.015
Public health focused subset	Accuracy	0.011^*^	[0.005, 0.017]	0.020
Public health focused subset	Macro F1	0.022^*^	[0.011, 0.033]	0.012
Public health focused subset	AUROC	0.015^*^	[0.007, 0.023]	0.017
Public health focused subset	AUPRC	0.034^*^	[0.018, 0.050]	0.009

Δ denotes the mean improvement of the proposed method over the strongest baseline. CI denotes the 95 percent confidence interval of the paired difference. The symbol ^*^ indicates *p* < 0.05 under a two sided paired *t* test.

#### 4.5.2 Illustrative application case for curriculum matrix construction

To further demonstrate how the proposed framework can be connected with educational practice, we provide an illustrative application case based on the test set outputs. This case is not intended to represent a completed institutional curriculum reform. Instead, it shows how the predicted learning effectiveness scores, event aligned representations, uncertainty estimates, and policy consistency signals can be translated into a curriculum matrix for teacher and curriculum committee interpretation. We use the OULAD test set as an illustrative example because it contains learner demographics, registration information, virtual learning environment interactions, assessment records, and final course outcomes. Following the experimental task definition, the model predicts the probability of course passing for each learner course instance. The weekly learning behavior records are aligned to relative course weeks, and the event aligned representations are used to identify weeks or modules in which learner engagement, assessment participation, or assignment completion becomes unstable. For curriculum interpretation, learners are first grouped according to their predicted passing probability. Instances with ŷ_*s*_ < 0.40 are treated as high risk cases, instances with 0.40 ≤ ŷ_*s*_ < 0.70 are treated as medium risk cases, and instances with ŷ_*s*_ ≥ 0.70 are treated as low risk cases. These thresholds are not used for model training; they are only used to support post hoc curriculum interpretation.

For each course week or instructional module, we summarize four types of model outputs: the average predicted learning risk level, the dominant event engagement pattern, the uncertainty level, and the policy consistency status. The learning risk level is derived from the predicted passing probability. The event engagement pattern is derived from the aligned weekly event representation and indicates whether the main difficulty is associated with weak platform activity, delayed participation, quiz instability, or assignment incompletion. The uncertainty level is derived from the uncertainty propagation component and reflects whether the available learning records are incomplete or noisy. The policy consistency status is derived from the policy regularization and event alignment constraints and indicates whether the observed learning pattern is consistent with curriculum requirements such as learning progression, participation balance, accessibility, and resource awareness.

Table 8 presents an example of the resulting curriculum matrix. The rows correspond to course weeks or modules, and the columns translate model outputs into curriculum interpretation and pedagogical action. For instance, if Week 3 shows a medium learning risk level, delayed participation, and moderate uncertainty, the curriculum team may consider adding a formative checkpoint or reminder activity before the corresponding assessment. If Week 6 shows a high learning risk level, weak quiz performance, and a policy consistency warning related to learning progression, the corresponding pedagogical action may include revising the prerequisite explanation, providing additional practice questions, or redistributing assessment difficulty. If Week 8 shows high uncertainty caused by unstable assignment records, the recommended action is not an immediate curriculum revision but a review of data completeness and feedback availability. This illustrative case shows how the proposed method can move from abstract model outputs to an interpretable curriculum support artifact. The curriculum matrix does not automatically prescribe curriculum reform. Instead, it organizes predictive, temporal, uncertainty related, and policy related evidence into a format that can be reviewed by teachers, curriculum designers, and institutional stakeholders. In practice, the final decision may include revising the sequence of instructional units, redistributing assessment workload, adding formative feedback, improving learning resource accessibility, or providing targeted support for learner groups with persistent risk. The proposed framework serves as a bridge between quantitative learning effectiveness prediction and pedagogical curriculum review. The illustrative curriculum matrix demonstrates how model outputs can be translated into curriculum review evidence, but it does not replace an institution level implementation study. Future work will collaborate with public health education programs to conduct institutional case studies and examine how teachers and curriculum committees use the proposed signals in real curriculum revision processes.

**Table 8:** Illustrative curriculum matrix generated from model outputs for curriculum decision support.

Course unit	Learning objective	Learning risk signal	Event engagement pattern	Uncertainty level	Policy consistency signal	Pedagogical interpretation and action
Week 1–2	Orientation and foundational concepts	Low	Stable access and regular activity	Low	Consistent with participation balance	Maintain the current structure and use the early activity pattern as a baseline for later monitoring.
Week 3–4	Core concept acquisition	Medium	Delayed participation and reduced active week continuity	Moderate	Mild warning on learning progression	Add a formative checkpoint, provide reminder messages, and offer short review materials before the next assessment.
Week 5–6	Quiz based formative assessment	High	Weak quiz performance and irregular assessment attempts	Moderate	Warning on learning progression and resource awareness	Review prerequisite explanation, provide additional practice questions, and adjust feedback timing for learners with weak assessment trajectories.
Week 7–8	Assignment completion and applied learning	High	Assignment incompletion and unstable submission behavior	High	Warning on accessibility and participation balance	Examine workload distribution, clarify assignment instructions, and provide targeted support or flexible feedback opportunities.
Week 9–10	Consolidation and final preparation	Medium	Recovery in platform activity but persistent uncertainty for some learners	Moderate	Generally consistent after support adjustment	Strengthen review sessions and monitor whether earlier interventions reduce predicted risk.

#### 4.5.3 Supplementary results on the public health focused HarvardX MITx subset

To strengthen the empirical connection between the proposed framework and public health curriculum optimization, a supplementary experiment is conducted on a public health focused subset constructed from the publicly available HarvardX MITx Person Course Academic Year 2013 De Identified Dataset. The full HarvardX MITx dataset is a large scale online learning dataset and is not exclusively limited to public health education. Therefore, learner course records are further filtered at the course level to form a more domain focused evaluation setting. Learner course records are retained when the corresponding course title, course code, or available course description is related to public health, population health, global health, environmental health, epidemiology, biostatistics, health systems, health policy, clinical research, or quantitative methods for health research. Records from unrelated courses are excluded from this subset. The prediction target follows the same definition as the full HarvardX MITx dataset. The system recorded certification variable is used as the binary course passing status, where certified learners are mapped to the positive class and non certified learners are mapped to the negative class. The same preprocessing pipeline, learner level data partition, baseline models, and evaluation metrics are used to ensure comparability with the original experiments. Table 9 reports the performance comparison on the public health focused HarvardX MITx subset. The proposed method achieves the best performance across all four metrics, with an Accuracy of 0.902, a Macro F1 of 0.763, an AUROC of 0.914, and an AUPRC of 0.682. Compared with the strongest baseline, FT Transformer, the proposed method improves Accuracy from 0.891 to 0.902, Macro F1 from 0.741 to 0.763, AUROC from 0.899 to 0.914, and AUPRC from 0.648 to 0.682. The results show that the proposed framework remains effective when the evaluation is restricted to public health related courses. The improvements in Macro F1 and AUPRC are especially important because certification outcomes in large scale online learning environments are usually imbalanced. These results indicate that policy aware indicator fusion, event driven temporal modeling, and uncertainty propagation provide complementary benefits for public health related course learning effectiveness prediction. The supplementary experiment provides more direct empirical evidence for the relevance of the proposed framework to public health curriculum decision support.

**Table 9:** Performance comparison on the public health focused HarvardX MITx subset.

Method	Accuracy	Macro F1	AUROC	AUPRC
LR	0.842 ± 0.006	0.641 ± 0.010	0.812 ± 0.009	0.512 ± 0.013
RF	0.861 ± 0.005	0.679 ± 0.008	0.846 ± 0.007	0.558 ± 0.011
LightGBM	0.879 ± 0.004	0.713 ± 0.007	0.875 ± 0.006	0.603 ± 0.010
LSTM	0.866 ± 0.007	0.698 ± 0.011	0.861 ± 0.010	0.579 ± 0.014
TabNet	0.884 ± 0.005	0.724 ± 0.009	0.887 ± 0.007	0.622 ± 0.012
FT transformer	0.891 ± 0.004	0.741 ± 0.008	0.899 ± 0.006	0.648 ± 0.010
Proposed method	**0.902 ± 0.003**	**0.763 ± 0.006**	**0.914 ± 0.005**	**0.682 ± 0.009**

Results are reported as mean and standard deviation over five repeated runs. The bolded values represent the optimal values.

#### 4.5.4 Ablation study

We conduct ablation studies to examine the contribution of the key modules in the proposed framework and to evaluate its sensitivity and robustness under controlled changes. The main ablation design removes one method component at a time while keeping the same learner course input variables, binary passing status label, datasets, preprocessing pipeline, train validation test partitions, and optimization settings. The evaluated datasets are OULAD and the HarvardX MITx Person Course Academic Year 2013 De Identified Dataset. The evaluation metrics follow the comparative experiments and include Accuracy, Macro F1, AUROC, and AUPRC. The ablated modules are selected from the method summary: policy constrained latent embedding, embedding reconstruction loss, policy regularization, event driven curriculum planning, uncertainty propagation, temporal participation policy alignment, feasibility manifold refinement, and uncertainty guided alignment refinement. This design tests whether each component contributes to predictive performance and whether the complete model provides complementary benefits beyond individual modules. For sensitivity analysis, we select the three most relevant hyperparameters for this method and task: the policy regularization coefficient λ, the uncertainty penalty coefficient γ, and the latent embedding dimension *d*_*z*_. Each sensitivity item is evaluated with two controlled values, producing a compact 2^3^ design. For robustness analysis, we evaluate one perturbation setting by randomly masking 10% of online learning behavior features in the test set. All ablation, sensitivity, and robustness experiments are repeated five times with matched random seeds.

Table 10 verifies that each component of the proposed framework contributes to the final prediction performance. The full model achieves 0.815 Macro F1 and 0.861 AUPRC on OULAD, and 0.798 Macro F1 and 0.764 AUPRC on HarvardX MITx. Removing the policy constrained latent embedding causes the largest degradation, reducing Macro F1 by 2.5 percentage points and AUPRC by 2.7 percentage points on OULAD, and reducing Macro F1 by 2.9 percentage points and AUPRC by 3.3 percentage points on HarvardX MITx. This confirms that the shared policy constrained manifold is essential for integrating heterogeneous learner, course, behavior, quiz, and assignment indicators. Removing temporal participation policy alignment also causes clear drops, from 0.815 to 0.794 Macro F1 on OULAD and from 0.798 to 0.774 on HarvardX MITx, indicating that event course mapping benefits from explicitly aligning temporal learning progress, participation patterns, and policy relevance. The variants without policy regularization and event driven curriculum planning perform worse than the full model, showing that policy consistency and weekly curriculum dynamics provide complementary information beyond static feature fusion. The smaller but consistent decline caused by removing uncertainty propagation and uncertainty guided alignment refinement demonstrates that uncertainty aware modeling mainly improves stability and robustness rather than only increasing average accuracy.

**Table 10:** Module level ablation results on OULAD and the HarvardX MITx Person Course Academic Year 2013 De Identified Dataset.

Variant	OULAD				HarvardX MITx			
	Acc.	Macro F1	AUROC	AUPRC	Acc.	Macro F1	AUROC	AUPRC
Full model	0.842 ± 0.003	0.815 ± 0.004	0.902 ± 0.003	0.861 ± 0.004	0.952 ± 0.001	0.798 ± 0.003	0.941 ± 0.002	0.764 ± 0.004
w/o policy constrained latent embedding	0.823 ± 0.005	0.790 ± 0.006	0.883 ± 0.005	0.834 ± 0.006	0.944 ± 0.002	0.769 ± 0.004	0.923 ± 0.003	0.731 ± 0.005
w/o Embedding reconstruction loss	0.833 ± 0.004	0.803 ± 0.005	0.893 ± 0.004	0.849 ± 0.005	0.948 ± 0.002	0.786 ± 0.004	0.934 ± 0.003	0.750 ± 0.005
w/o policy regularization	0.828 ± 0.004	0.797 ± 0.005	0.889 ± 0.004	0.842 ± 0.005	0.946 ± 0.002	0.778 ± 0.004	0.929 ± 0.003	0.742 ± 0.005
w/o event driven curriculum planning	0.831 ± 0.004	0.800 ± 0.005	0.891 ± 0.004	0.846 ± 0.005	0.947 ± 0.002	0.782 ± 0.004	0.931 ± 0.003	0.746 ± 0.005
w/o uncertainty propagation	0.835 ± 0.004	0.805 ± 0.005	0.895 ± 0.004	0.852 ± 0.005	0.949 ± 0.002	0.788 ± 0.004	0.935 ± 0.003	0.752 ± 0.005
w/o temporal participation policy alignment	0.826 ± 0.005	0.794 ± 0.006	0.886 ± 0.005	0.839 ± 0.006	0.945 ± 0.002	0.774 ± 0.004	0.926 ± 0.003	0.737 ± 0.005
w/o feasibility manifold refinement	0.830 ± 0.004	0.799 ± 0.005	0.890 ± 0.004	0.845 ± 0.005	0.947 ± 0.002	0.781 ± 0.004	0.930 ± 0.003	0.745 ± 0.005
w/o uncertainty guided alignment refinement	0.837 ± 0.004	0.808 ± 0.005	0.897 ± 0.004	0.855 ± 0.005	0.950 ± 0.002	0.791 ± 0.004	0.937 ± 0.003	0.756 ± 0.005

Results are reported as mean ± standard deviation over five runs.

Table 11 further shows that the proposed method is not overly sensitive to a narrow hyperparameter choice. The best configuration is λ = 0.10, γ = 0.010, and *d*_*z*_ = 128, which matches the full model setting and obtains 0.815 Macro F1 and 0.861 AUPRC on OULAD, as well as 0.798 Macro F1 and 0.764 AUPRC on HarvardX MITx. Reducing the latent dimension to *d*_*z*_ = 64 lowers Macro F1 by 0.4–1.4 percentage points, suggesting that a larger latent space better preserves multi source curriculum indicators. Under 10% behavior feature masking, the model still obtains 0.803 Macro F1 and 0.848 AUPRC on OULAD, and 0.786 Macro F1 and 0.747 AUPRC on HarvardX MITx. Compared with the clean full model in Table 10, the degradation remains moderate, supporting the robustness of the proposed uncertainty propagation and uncertainty guided alignment mechanisms when online behavior logs are incomplete or noisy.

**Table 11:** Sensitivity and robustness results on OULAD and the HarvardX MITx Person Course Academic Year 2013 De Identified Dataset.

Setting	OULAD		HarvardX MITx	
	Macro F1	AUPRC	Macro F1	AUPRC
λ = 0.05, γ = 0.005, *d*_*z*_ = 64	0.801 ± 0.005	0.846 ± 0.005	0.783 ± 0.004	0.746 ± 0.005
λ = 0.05, γ = 0.005, *d*_*z*_ = 128	0.807 ± 0.004	0.854 ± 0.005	0.790 ± 0.004	0.755 ± 0.005
λ = 0.05, γ = 0.010, *d*_*z*_ = 64	0.806 ± 0.005	0.853 ± 0.005	0.789 ± 0.004	0.753 ± 0.005
λ = 0.05, γ = 0.010, *d*_*z*_ = 128	0.811 ± 0.004	0.858 ± 0.004	0.794 ± 0.003	0.760 ± 0.004
λ = 0.10, γ = 0.005, *d*_*z*_ = 64	0.809 ± 0.004	0.856 ± 0.004	0.792 ± 0.003	0.758 ± 0.004
λ = 0.10, γ = 0.005, *d*_*z*_ = 128	0.812 ± 0.004	0.859 ± 0.004	0.795 ± 0.003	0.761 ± 0.004
λ = 0.10, γ = 0.010, *d*_*z*_ = 64	0.811 ± 0.004	0.858 ± 0.004	0.794 ± 0.003	0.760 ± 0.004
λ = 0.10, γ = 0.010, *d*_*z*_ = 128	0.815 ± 0.004	0.861 ± 0.004	0.798 ± 0.003	0.764 ± 0.004
10% behavior feature masking	0.803 ± 0.005	0.848 ± 0.006	0.786 ± 0.004	0.747 ± 0.006

Results are reported as mean ± standard deviation over five runs.

## 5 Discussion

### 5.1 Ethical and political considerations

The proposed framework supports curriculum related decision making under policy aware constraints, and therefore its ethical and political implications require explicit discussion. Curriculum is not a neutral technical object. It involves decisions about what knowledge should be prioritized, which learner needs should receive attention, how institutional resources should be allocated, and whose perspectives are represented in the curriculum. In public health education, these questions are especially significant because curriculum decisions are connected with equity, community preparedness, professional responsibility, and the distribution of health related knowledge. Data driven curriculum optimization should not be understood as an automatic replacement for educational, political, or pedagogical deliberation.

A first ethical risk is the technocratization of curriculum. If model outputs are treated as direct prescriptions, curriculum design may be reduced to predicted passing probabilities, measurable indicators, and optimization objectives. Such a reduction would marginalize teacher judgment, learner experience, contextual knowledge, and democratic debate. To avoid this risk, the proposed framework should be used as a decision support tool rather than as an automatic curriculum authority. The predicted learning effectiveness scores, event aligned representations, uncertainty estimates, and policy consistency signals are intended to support reflection and discussion, not to determine curriculum revisions without human interpretation. A second concern is the possible reinforcement of structural inequalities. Learner course records may contain socio demographic, behavioral, and assessment variables that reflect unequal access to learning time, digital infrastructure, prior academic preparation, language resources, or institutional support. If these indicators are interpreted uncritically, the model may reproduce existing inequalities by treating historically disadvantaged learner groups as inherently high risk. Risk predictions should not be used for exclusion, tracking, punitive intervention, or lowering expectations for particular groups. Instead, they should be interpreted as evidence for additional support, accessibility improvement, resource redistribution, and pedagogical adjustment. In this sense, policy aware constraints should be designed and reviewed to promote equity, participation balance, and learning opportunity rather than merely to improve aggregate predictive performance. A third issue concerns the political nature of public health curriculum priorities. Public health education often involves contested questions, including the balance between individual responsibility and collective welfare, the role of institutions in health protection, the distribution of preventive resources, and the relationship between scientific expertise and community experience. These questions cannot be resolved by predictive modeling alone. A model may identify where learning risks or participation discontinuities occur, but it cannot determine which public health values should guide curriculum reform. Such decisions require deliberation among teachers, learners, curriculum committees, public health professionals, institutional administrators, and, where appropriate, community stakeholders. A fourth concern is democratic accountability in automated decision making. Model based recommendations should remain transparent, reviewable, contestable, and revisable. Curriculum stakeholders should be able to understand which indicators contribute to a recommendation, how uncertainty affects the reliability of the output, and whether policy consistency signals reflect agreed educational values. The curriculum matrix proposed in this study is therefore intended as an interface for deliberation rather than as a final decision. It organizes learning risk signals, engagement patterns, uncertainty levels, and policy related warnings into a form that can be examined by educators and institutional stakeholders.

To support responsible use, several safeguards are necessary. Model outputs should be accompanied by uncertainty estimates and should not be used as the sole basis for high stakes curriculum or learner level decisions. Subgroup level performance and error patterns should be monitored to examine whether the model disproportionately affects particular learner groups. The design of policy constraints should be documented and periodically reviewed by curriculum and policy stakeholders. Learners and teachers should have opportunities to question and contextualize model based recommendations. Curriculum decisions informed by the model should be recorded together with the human reasoning used to accept, reject, or revise the recommendation. These safeguards help ensure that policy aware curriculum optimization remains aligned with educational responsibility, equity, and democratic governance.

### 5.2 Cultural awareness and policy determinization

Another important limitation concerns cultural awareness and the risk of policy determinization. Although the proposed framework is context aware in the sense that it incorporates learner course indicators, course context, participation patterns, and policy related constraints, it should not be interpreted as fully culturally aware. Cultural identity, community experience, language background, local trust in public health institutions, and culturally specific understandings of health cannot be completely represented by standard socio demographic variables or platform based learning records. If these dimensions are not explicitly included in the design of policy constraints, qualitative review, or stakeholder deliberation, they may remain invisible to the model and therefore absent from the curriculum support output. This issue reflects a broader limitation of mathematically driven policy optimization. Pre defined policy constraints can help prevent purely performance driven prediction, but they also determine in advance which policy values are visible to the model. As a result, the optimization process may favor measurable objectives such as passing probability, participation frequency, assessment completion, or resource efficiency, while underrepresenting less easily measurable but educationally important concerns such as cultural safety, learner belonging, community relevance, epistemic diversity, and trust. Policy aware optimization should not be understood as a complete representation of public health values. It is a formal decision support mechanism whose scope is limited by the indicators, constraints, and stakeholder assumptions used to construct it. To reduce this risk, the policy constraint set should be treated as a revisable and participatory component rather than as a fixed technical input. Curriculum designers should review whether the selected indicators and constraints adequately reflect the cultural and social contexts of the learners and communities served by the public health curriculum. Where appropriate, qualitative evidence, learner feedback, teacher interpretation, and community or stakeholder consultation should be used to revise the policy constraints and to contextualize model outputs. In particular, high predicted effectiveness should not be interpreted as evidence that a curriculum is culturally inclusive or socially responsive. Conversely, low predicted effectiveness for a learner group should not be attributed to cultural identity itself, but should prompt examination of whether the curriculum, language, support structure, assessment design, or institutional conditions are insufficiently responsive to that group.

## 6 Conclusions and future work

In this study, we investigated public health curriculum optimization from a data driven and policy aware perspective. We formulated curriculum optimization as a supervised learning effectiveness prediction problem, where course passing status serves as an operational proxy for successful learning outcomes. Based on structured learner course indicators and weekly learning records, we proposed the *Policy Driven Indicator Integrator*, a unified framework that integrates heterogeneous learner indicators and policy related constraints into a shared modeling space. The framework consists of three core components: the *Manifold Constrained Optimizer*, which learns policy consistent latent representations; the *Event Driven Curriculum Planner*, which captures weekly learning dynamics and event engagement patterns; and the *Uncertainty Propagation Forecaster*, which improves robustness under noisy and incomplete learner course records. Together with the *Event Segmentation Alignment* strategy, the proposed framework aligns temporal, participation, accessibility, resource, and policy dimensions, thereby transforming curriculum related decision support into a quantitatively evaluable and interpretable modeling task.

Experimental results on OULAD and the HarvardX MITx Person Course dataset demonstrate that the proposed method consistently outperforms strong baselines, including LightGBM, TabNet, and FT Transformer, across multiple metrics such as Macro F1 and AUPRC. These results indicate that the policy constrained latent embedding can effectively capture higher order relationships among educational, socio demographic, behavioral, assessment, and course context features, while the event driven and uncertainty aware components provide complementary gains in modeling temporal learning dynamics and data uncertainty. The efficiency analysis further shows that the proposed method achieves a favorable trade off between predictive performance and computational cost, making it suitable for practical deployment in data supported curriculum optimization scenarios. More importantly, the model outputs can be translated into curriculum review evidence, including learning risk signals, event engagement patterns, uncertainty levels, and policy consistency information, which can support teachers, curriculum designers, and policy stakeholders in reflective and evidence informed curriculum decision making.

Despite these advantages, several limitations remain. The current framework relies on predefined policy related constraints, which may not fully capture rapidly evolving educational policies, institutional requirements, public health priorities, or culturally situated learning experiences. Although socio demographic and course context indicators are included, they cannot fully represent cultural identity, cultural safety, community trust, epistemic diversity, or the broader values embedded in public health curriculum design. In addition, the event segmentation and alignment procedure introduces additional computational overhead, especially for large scale datasets with dense temporal interactions. Future work will explore adaptive policy modeling mechanisms that update constraints based on streaming learner course data, external policy signals, and stakeholder feedback. It will also improve the scalability of temporal alignment modules and extend the framework from learning effectiveness prediction to curriculum recommendation, intervention planning, and real world decision support. At the same time, qualitative curriculum analysis, teacher interpretation, participatory policy constraint design, learner feedback, and community stakeholder consultation should be further integrated to strengthen the dialogue between data driven modeling and educational theory. The proposed model should be understood not as an automatic curriculum prescription mechanism, but as a decision support tool for pedagogical reflection, democratic accountability, and culturally responsive public health education.

## Data Availability

The original contributions presented in the study are included in the article/supplementary material, further inquiries can be directed to the corresponding author.
